# Fate of *in vitro* cultured *Mycobacterium abscessus* populations when exposed to moxifloxacin

**DOI:** 10.3389/fmicb.2024.1494147

**Published:** 2024-11-28

**Authors:** Elena G. Salina, Billy A. Martini, Vladimir V. Sorokin, Andrey L. Mulyukin

**Affiliations:** ^1^Bach Institute of Biochemistry, Research Center of Biotechnology of the Russian Academy of Sciences, Moscow, Russia; ^2^Shemyakin and Ovchinnikov Institute of Bioorganic Chemistry of the Russian Academy of Sciences, Moscow, Russia; ^3^Winogradsky Institute of Microbiology, Research Center of Biotechnology of the Russian Academy of Sciences, Moscow, Russia

**Keywords:** *Mycobacterium abscessus*, moxifloxacin, antimicrobials, bactericidal activity, survivors, drug insensitive subpopulation

## Abstract

Given the current need for predictive persisting model for *Mycobacterium abscessus*, we adopted a classical assay to study drug-tolerant bacterial persisters, focusing on the behavior of a small antibiotic-insensitive subpopulation during prolonged exposure to moxifloxacin. Our study showed a wide-ranging response of *M. abscessus*, depending on antibiotic concentration, growth stage of mycobacterial cultures, and the availability of potassium ions in the medium. Mid-logarithmic cultures, initially grown in either balanced or K^+^-free medium, contained small sup-populations capable of prolonged and stable survival in the presence of moxifloxacin. The response of these mid-log cultures to antibiotic exposure involved initial killing, followed by regrowth at 1–2 MBCs of moxifloxacin or a substantial reduction of the antibiotic-insensitive subpopulation to fewer than 10^2^ CFU/mL at 16 MBCs. In stationary-phase cultures grown in a complete medium, a consistent number of viable cells was observed when exposed to a high dose of moxifloxacin. In contrast, antibiotic-insensitive subpopulations in stationary-phase *M. abscessus* cultures under potassium-deficient conditions experienced gradual killing across a wide range of moxifloxacin concentrations (1–16 MBCs). Studies on electron microscopy demonstrated that singular cells were rapidly destroyed after relatively short-term exposure to moxifloxacin, while cells in aggregates or clumps persisted longer, explaining the delayed biocidal effect. The small subpopulation that survived under intense moxifloxacin pressure was notably heterogeneous in cell morphology and fine structure, consisting of ovoid forms and cell-wall-deficient cells with reduced size. These findings suggest that the same antibiotic dose may have varying effects on *M. abscessus* cells, depending on their physiological state and abundance within infected cells or tissues. Taken together, our study may contribute to the development of strategies to combat recalcitrant survivor subpopulations.

## 1 Introduction

*Mycobacterium abscessus* can cause severe lung, skin, soft tissue, and mucosal infections, whose incidences are constantly increasing, especially in patients with underlying respiratory diseases, particularly cystic fibrosis, bronchiectasis, chronic obstructive pulmonary disease, and so on (Bryant et al., 2021; Degiacomi et al., 2019; Cambau et al., 2024). *M. abscessus* is a critical target in the search for new drugs and treatment strategies, given its high resistance to most currently used antibiotics, including macrolides, aminoglycosides, rifamycins, tetracyclines, and β-lactams (Nessar et al., 2012; Recchia et al., 2023; Fröberg et al., 2023). The major role in the intrinsic resistance of mycobacteria to antimicrobial agents is played by the low permeability of the *M. abscessus* cell wall, nucleotide variants in genes coding for drug targets, numerous enzymes neutralizing antibiotics, a lack of drug-activating enzymes, induction of drug efflux pumps, and so on (Luthra et al., 2018; Johansen et al., 2020).

Extended antibiotic treatment may result in acquired drug resistance due to modifications of the genes encoding specific drug targets (Bastian et al., 2011; Maurer et al., 2012; Prammananan et al., 1998).

Besides genetically determined mechanisms of resistance, there is another phenomenon of drug insusceptibility: nonheritable antibiotic tolerance or bacterial persistence. The phenomenon of bacterial persistence was first noticed in the 1940s upon experiments with penicillin, showing that 1% of pathogenic bacteria were found to survive after treatment (Hobby et al., 1942). Later, Bigger, who studied this phenomenon in more detail, called a small number of staphylococci that survived exposure to penicillin “persisters” (Bigger, 1944). Persister cells are a small subpopulation that can withstand lethal concentrations of bactericidal antibiotics without developing resistance (Balaban et al., 2019; Newson et al., 2022) and emerge upon antibiotic withdrawal, causing the resumption of infection (Swaminath et al., 2020; Lewis, 2007). Notably, persisters isolated from an infected animal retain their virulence upon reinfection (Newson et al., 2022). Moreover, phenotypic heterogeneity of bacterial populations with the presence of a subpopulation of drug-tolerant persisters is recognized as one of the virulence strategies of the pathogen (Weigel and Dersch, 2018; Newson et al., 2022; Shang et al., 2020).

Drug-tolerant persisters, irrespective of broad or traditional definitions of the persistence phenomenon (Balaban et al., 2019; Gold and Nathan, 2017), are recognized targets in the discovery of potent drugs against various pathogenic bacteria (Lewis, 2007; Wainwright et al., 2021; Niu et al., 2024; Lewis, 2012). As with *M. abscessus*, the emerging and resistant pathogen that has attracted great attention in repurposing the existing and discovery of new antimicrobial agents (Nguyen et al., 2024), it is currently topical to adopt and implement persister-specific assays (Wu et al., 2018). While the phenomenon of bacterial persistence is well-studied, the antibiotic persistence of the emerging pathogen *M. abscessus* remains poorly understood. Non-growing starved cells in phosphate buffer were used in addition to conventionally tested planktonic cells in aerated rich medium (Berube et al., 2018). The most comprehensive assay encompassed biofilms, non-replicating cells in nutrient-starved or anaerobic cultures for MIC and MBC determinations (Yam et al., 2020). In this study, we employed the classical methodology and approaches used for the other bacteria (Balaban et al., 2004; Keren et al., 2004; Lewis, 2007) with a focus on a typically small drug-insensitive subpopulation in mid-logarithmic and stationary-phase *M. abscessus* cultures that could survive and resume growth after antibiotic withdrawal.

We hypothesize that *M. abscessus* response to antibiotics depends on the growth stage, the medium composition, the concentration of an antimicrobial agent, and a relatively stable growth-arrest state that occurs under certain experimental conditions. The bi- or triphasic response to prolonged exposure to antibiotics, involving the successive killing, persistence, and regrowth stages, has been known for the other mycobacteria—*M. tuberculosis* and *M. smegmatis* (Sebastian et al., 2017; Swaminath et al., 2020) and is expected for *M. abscessus*. Since susceptibilities of *M. abscessus* to antimicrobials varied greatly for aerated, stressed, hypoxic cultures (Yam et al., 2020), it is impossible to predict the behavior of a subpopulation developing under different growth and nutritional conditions and left viable after killing the antibiotic-sensitive majority. Using viability tests and a combination of electron microscopy methods, we show that the fate of *M. abscessus* under prolonged exposure to moxifloxacin, a recommended antibiotic, is conditional upon the physiological state of cultures prior to the antibiotic challenge. For this study, we specifically selected potassium deficiency in the growth media as a stress condition, which promotes the transition of cultures to a metabolically inactive state with a significant loss of colony-forming ability, as shown for *M. abscessus* (Mulyukin et al., 2023) and *M. tuberculosis* (Salina et al., 2014a,b).

## 2 Materials and methods

### 2.1 Bacterium and media

*Mycobacterium abscessus* ATCC 19977^T^ was provided by the European Polytechnic School of Lausanne (Lausanne, Switzerland) and stored at −70°C. Starter cultures were initially grown from frozen stocks in 7H9 (Himedia, India) with 10% ADS (0.5% BSA, 0.2% dextrose, 0.085% sodium chloride) and 0.05% Tween-80 (Neofroxx GmbH, Germany) at 37°C with shaking at 200 rpm for 3 days. A starter culture was inoculated (0.25%) into Sauton medium, containing: KH_2_PO_4_, 0.5 g; MgSO_4_·7H_2_O, 1.4 g; L-asparagine, 4 g; glycerol, 60 mL; ferric ammonium citrate, 0.05 g; sodium citrate, 2 g; 1% ZnSO_4_ · 7H_2_O, 0.1 mL; H_2_O, to 1 L; pH 7.0 (adjusted with 1 M NaOH) or into potassium-free Sauton media with ADS and Tween-80 in which K^+^ ions (3.7 mM) were equimolarly substituted for Na^+^ ions (Salina et al., 2014a,b) with the addition of 10% ADS and 0.05% Tween-80. Cultures were incubated in loose-capped flasks at 37°C with shaking at 200 rpm for 3 days to the mid-log phase or 10–14 days to the stationary phase.

### 2.2 Minimum inhibitory concentration

Determination of antibiotic MICs using the resazurin reduction microplate assay (REMA) was performed as previously described (Palomino et al., 2002). Briefly, a series of consequential twofold dilutions of amikacin, bedaquiline, ciprofloxacin, clofazimine, linezolid, moxifloxacin, or rifampicin in Sauton medium with 10% ADS was added with 100-μl aliquots to wells in Corning plates (Corning, USA) in the concentration range from 64 to 0.5 μg/mL. Middle-logarithmic *M. abscessus* cultures in Sauton medium were diluted with fresh medium to 2ˑ10^5^ CFU/mL and then added in 100-μL portions to each well with and without antibiotics (control) to final 200-μL volumes and 1ˑ10^5^ CFU/mL. After incubation of the plates for 24 h at 37°C, resazurin (Merck, Germany) was added to wells at the concentration of 0.025 mg/mL. Following overnight incubation at 37°C, the fluorescence of resorufin, the resazurin metabolite, was measured using a Fluostar Omega microplate reader (BMG-Labtech, Germany) at the excitation and emission wavelengths of 544 nm and 590 nm, respectively. The MIC was defined as the lowest concentration that prevented resazurin from turning blue to pink, based on both visual observation and fluorescence measurements (Lechartier et al., 2012).

### 2.3 Bactericidal effect

*Mycobacterium abscessus* cells were grown in Sauton medium to either the mid-log or stationary phase (Supplementary Figure 1), sampled into sterile Falcon tubes (Corning, USA), and pelleted by centrifugation at 3000 × *g* for 15 min. The supernatants from each culture were transferred to separate tubes. Pelleted mid-log cells were resuspended using the supernatant from mid-log phase cultures, while stationary-phase cells were resuspended with the supernatant from stationary-phase cultures. Fresh Sauton medium or buffer was not used to prevent further growth or starvation stress.

Suspensions were adjusted to a cell density of OD_600_ = 0.1, agitated at 100 rpm at 37°C for approximately 4 h (a period shorter than the mean generation time of 5 h), and treated with 31, 63, 125, 250, and 500 μg/mL of moxifloxacin (Merck, Germany). The cultures were incubated for 1, 3, 7, and 14 days (at 37°C with agitation at 100 rpm). Mid-log cultures were further diluted with the corresponding supernatant to OD_600_ = 0.001 (~5 ˑ10^5^ CFU/mL) and OD_600_ = 0.00001 (~5 ˑ10^3^ CFU/mL) and exposed to 4 and 10 μg/mL of moxifloxacin for 1, 2, 3, 5, and 7 days (at 37°C with agitation at 100 rpm).

After antibiotic treatment, cultures were washed with fresh Sauton or potassium-free Sauton media supplemented with ADS and Tween-80 to remove residual antibiotics. Then, the bactericidal effect was evaluated using CFU and MPN assays.

### 2.4 Viability tests

Tenfold serially diluted cell suspensions were plated in triplicates onto 7H10 agar plates (Himedia, India) supplemented with 10% ADS in *Petri dishes* and incubated at 37°C for 6 days, followed by CFU counting. The bactericidal effect was also evaluated using the most probable number (MPN) method to account for cells that had lost the ability to form CFU on agar plates but could still grow in a liquid medium. For the MPN assays, the same tenfold serial dilutions were inoculated into diluted liquid Sauton medium (1:1 [vol/vol]; final concentration of glycerol, 0.6%) (Salina et al., 2014a,b), supplemented with 10% ADS, in 96-well Corning plates. The plates were left to stand at 37°C for 8–10 days. Wells with visible bacterial growth were counted as positive, and MPN values were calculated with 95% confidence limits using statistical tables designed based on probability histograms (de Man, 1975). MPN values were calculated by counting wells with visible turbidity for a series of at least five serial tenfold dilutions prepared in triplicate. The exact confidence limits, with a minimal probability of 95%, for each MPN value were obtained from the corresponding statistical tables (de Man, 1975).

### 2.5 Transmission electron microscopy (TEM)

Cells from 10-mL cultures were harvested upon pelleting down at 4000 *g* for 10 min and washed twice in sterile mQ water with further centrifugation. Then, cells were fixed in 2.5% glutaraldehyde (w/v) in 0.1 M sodium cacodylate buffer (pH 7.2) for 2.5 h, pelleted, and then post-fixed in 1% (w/v) osmium tetroxide in the same buffer for 12 h. The osmium fixative was removed upon centrifugation, and a heated sterile water solution with agar (2%) was added dropwise to the residue and allowed to stay for solidification. The agar-embedded material was cut into millimeter-sized pieces and dehydrated in 3% uranyl acetate in 30% ethanol for 2 h and 70% ethanol for 12 h with further 10-min changes in a series: 96% ethanol (twice); 96%-acetone (1: 1 v/v) and acetone (trice). Dehydrated specimens were soaked with epoxy resin Epon 812 (Fluka, Switzerland) with the components—acetone mixtures (1:1 and 1:2 v/v)—and placed in capsules with the resin (without acetone) for polymerization at 40°C for 24 h and then 60°C for 24 h. The produced blocks were trimmed across visible sediment using an 8,800 Ultrotome III (LKB-Produkter, Sweden) to prepare thin sections. Sections were placed on copper grids (Jeol, Tokyo, Japan) coated with Formvar and stained with aqueous 3% uranyl acetate and then 3.5% lead citrate for 20 min at 37°C, and air-dried for 24 h. Specimens were examined under a JEM-1400 electron microscope (Jeol, Japan) operating at 80 kV.

### 2.6 Scanning electron microscopy (SEM)

Starting and 10-fold diluted suspensions of cells after washing in mQ water were dropped on cleaned coverslip pieces mounted on SEM specimen stubs. After drying in the air for 12 h in Perti dishes, the samples were coated with Au in a JFC-1100 ion sputter (Jeol, Tokyo, Japan). Specimens were examined under a JSM-IT200 electron microscope (Jeol, Japan).

### 2.7 Energy dispersive X-ray spectroscopy (EDX)

Harvested cells were washed from the medium with distilled water, re-suspended in different dilutions, and dropped with 5–10-μL aliquots on Formvar-coated and carbon-reinforced copper grids and air-dried for 12 h (Washing of cells was necessary to prevent film formation from medium components; fixatives were inadmissible to alter the elemental composition). Specimens were subjected to EDX spectroscopy analysis under TEM mode with recording of total images and mapping of elements using a JEM-1400 microscope (Jeol, Japan) equipped with an energy-dispersive X-ray analysis system (EDXA, Inca Energy-350, Oxford Instruments, UK), operating at an accelerating voltage of 80 keV (tilt angle, 15°). The examination procedure involved choosing a TEM image and EDX spectroscopy to detect all chemical elements in the entire image or the region of interest with mapping of all or optionally selected elements using Aztec software (Oxford Instruments, UK). A map for each element was automatically marked with different colors for visual representation. Electron microscopy studies were performed in the UNIQEM Collection Core Facility.

### 2.8 Analysis of SEM and TEM images

Suitable SEM and TEM images were used for morphometric analysis to estimate quantitatively the heterogeneity of populations in the cell size and morphological types in control or experimental groups. We examined SEM micrographs and distinguished between individual cells with intact, destructed, thinned, and smoothened surfaces, counted their numbers, and calculated their proportions. The mean length and width of intact were measured on TEM images of longitudinal thin sections. Suitable transverse sections across the long axis were also used to calculate the mean width; ‘empty’ sections were excluded from the analysis. Cells were referred to the N category (as those in the corresponding control group) if their dimensions were close to 1.70 ± 0.36 μm × 0.34 ± 0.07 μm (L × W); cells with more than twice shorter length and/or width were referred to the reduced size category (R category). Upon examinations of TEM images, we ascribed individual cells to a particular morphological type and evaluated their occurrence relative to the numbers of all or intact cells. TEM and corresponding EDX spectral maps were pairwise compared to confirm the presence/absence of elements in cells viewed on TEM images. In particular, cells on TEM images were grouped into clumps (aggregates) and singular cells and then into potassium-containing and free subgroups.

### 2.9 Statistics

Experiments were conducted in at least two biological and three technical replications. Statistical analysis was performed using Microsoft^®^ Office^®^ Excel 2016 MSO (16.0.4639.1000). The data were expressed as the mean ± standard deviation. Data were analyzed using Student’s unpaired *t*-test; a *p-*value of < 0.05 was considered statistically significant.

## 3 Results

### 3.1 Insufficient killing activity of moxifloxacin at 2 and 5 MICs against *Mycobacterium abscessus*

Traditionally, the minimum inhibitory concentration (MIC) is defined as the lowest drug concentration that prevents a bacterial suspension of 1ˑ10^5^–5 ˑ10^5^ CFU/mL from becoming turbid after a defined incubation period. The minimum bactericidal concentration (MBC) is determined as the lowest drug concentration that reduces the bacterial population with 1ˑ10^5^–5ˑ10^5^ CFU/mL to 1ˑ10^2^–5ˑ10^2^ CFU/mL during the incubation period, but it has been proposed to lower this threshold to 99.0% for slowly growing mycobacteria (Maurer et al., 2014; Greendyke and Byrd, 2008). We used different initial concentrations of cells for primary assays of moxifloxacin activity against *M. abscessus* for the following reasons.

In addition to the previously initial cell densities, a starting concentration of 5 ˑ10^7^ CFU/mL was used because the residual subpopulation after moxifloxacin exposure could not be reliably quantified using CFU assays at lower densities (5 ˑ10^5^ CFU/mL) Suspensions with 5 ˑ10^3^ CFU/mL were used to assess responses to moxifloxacin, considering that mycobacteria may exist in microenvironments with low cell densities.

**Figure 1:**
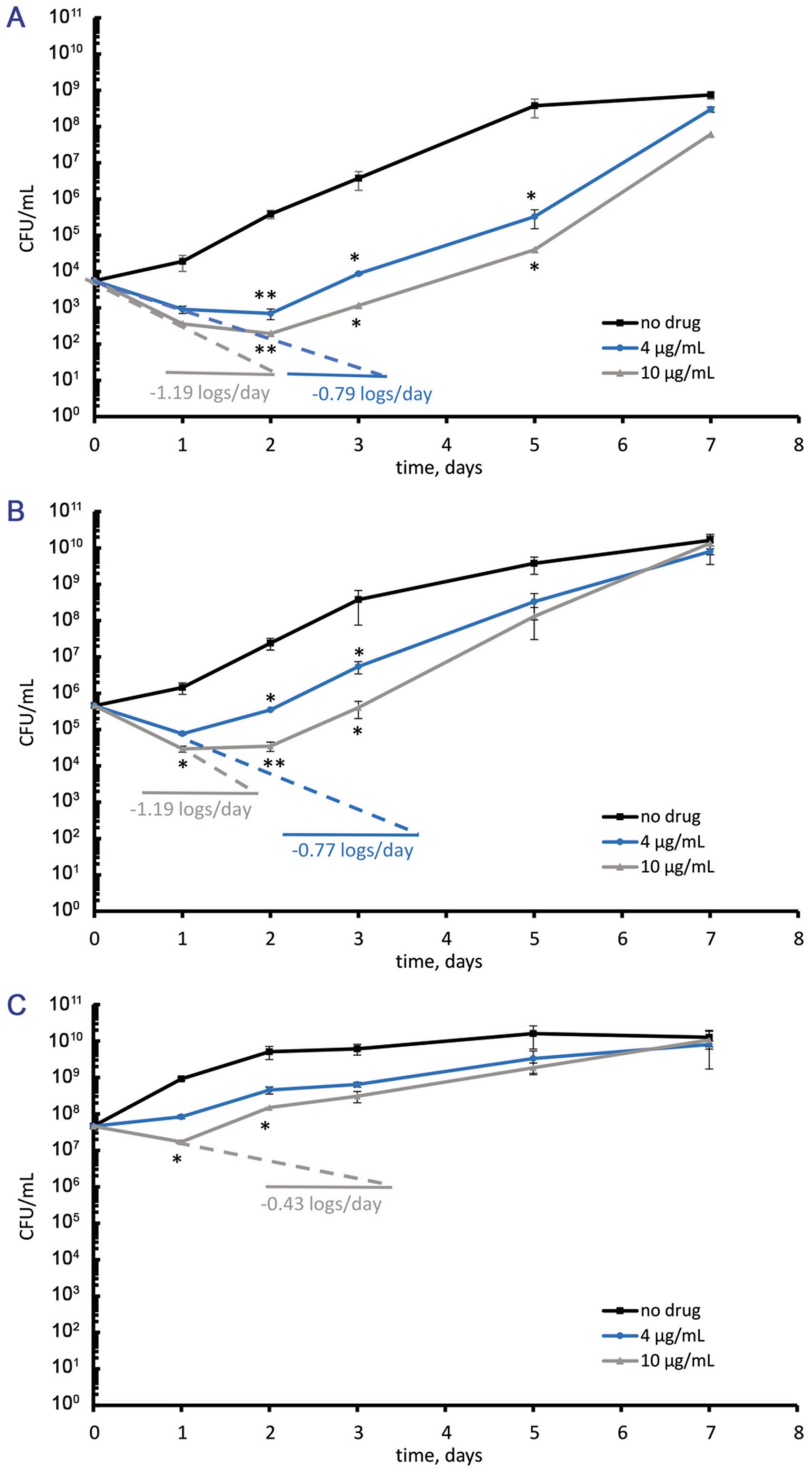
Numbers of viable cells (CFU/mL) in *M. abscessus* suspensions grown to the mid-logarithmic phase, diluted with the collected supernatant to various cell densities, and incubated in the absence or the presence of moxifloxacin added at concentrations of 4 and 10 μg/mL (2 and 5 MICs, respectively). **(A)** Initial cell density ~ 5ˑ10^3^ CFU/mL. **(B)** Initial cell density ~ 5ˑ10^5^ CFU/mL (as in conventional assays for MBC). **(C)** Initial cell density ~ 5ˑ10^7^ CFU/mL. The experiments were performed independently twice in triplicates; the mean values and standard deviations are shown here: *p < 0.05, **p < 0.01, unpaired *t*-test.

For this study, MIC determinations for amikacin, bedaquiline, ciprofloxacin, clofazimine, linezolid, moxifloxacin, and rifampicin were conducted using the microtiter rezazurin assay (Supplementary Table 1). Bedaquiline and moxifloxacin with MICs of 2 μg/mL appeared to be the most active among the rest of the antibiotics (Supplementary Table 1). Unlike bedaquiline, moxifloxacin exerted activity against both actively growing and ‘non-culturable’ metabolically inert *M. abscessus* cells (Mulyukin et al., 2023) and was chosen for further experiments reported below. The MBC of moxifloxacin exceeded 5 MICs (10 μg/mL), as found from killing curves for cell suspensions at the same cell density of ~5ˑ10^5^ CFU/mL. In this case, treatment with 2 and 5 MICs of moxifloxacin resulted in a decrease of CFU numbers (<2 log_10_ units/day) with the subsequent regrowth to the cell numbers as in the control antibiotic-free culture (Figure 1B). *M. abscessus* suspensions that were adjusted to a low density (~5ˑ10^3^ CFU/mL) upon dilution with the collected cell-free supernatant displayed a similar response, and the MBC was higher than 10 μg/mL (Figure 1A). Subsequent experiments excluded suspensions with 10^3^–10^5^ CFU/mL of *M. abscessus*, as the small fractions of surviving cells were not reliably detectable using CFU assays.

Mid-log *M. abscessus* cells at the elevated cell density of ~5ˑ10^7^ CFU/mL (OD_600_ = 0.1) responded to 5 MICs of moxifloxacin (10 μg/mL) with a 60% reduction in the CFU numbers within 24 h followed by the regrowth during the next 6 days (Figure 1C). Two MICs (4 μg/mL) of moxifloxacin caused a bacteriostatic effect, as seen from unchanged CFU numbers over 24 h, which was followed by regrowth to the cell density as in control antibiotic-free cultures (Figure 1C). It is noteworthy that exposure to moxifloxacin (2 or 5 MICs) for 24 h was insufficient to abolish antibiotic-sensitive subpopulations. Regarding the relatively slow and incomplete or inefficient killing of active *M. abscessus* cells upon exposure to moxifloxacin in several MICs (Figure 1), we extended the antibiotic concentration range to reveal low-abundant surviving fractions.

### 3.2 The MBCs of moxifloxacin for *Mycobacterium abscessus* grown in complete or potassium-free Sauton media

The MВС for mid-log *M. abscessus* cultures (initial density ~ 5ˑ10^7^ CFU/mL; OD_600_ = 0.1) grown in each medium (Supplementary Figure 1) was 31 μg/mL, as determined by CFU counts (Supplementary Figures 2A,C). Similarly, the MBC after 3 days of exposure to moxifloxacin for stationary-phase *M. abscessus* cells (initial density ~ 5ˑ10^7^ CFU/mL; OD_600_ = 0.1) grown in either complete or potassium-free medium (Supplementary Figure 1) was also 31 μg/mL (Supplementary Figures 3A,C). However, the bactericidal effect, indicated by a CFU reduction of more than 2 log_10_ units, varied in duration depending on the medium used for cultivation (Supplementary Figures 2, 3).

**Figure 2:**
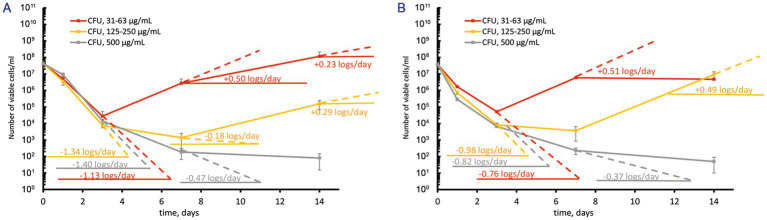
Response of *M. abscessus* cultures grown to the mid-logarithmic phase to increasing over-MBC moxifloxacin concentrations. The graphs show the corresponding groups with statistically significant differences (*p* < 0.05). **(A,B)** The CFU/ml changes for cultures grown in the complete or potassium-free Sauton media, respectively.

**Figure 3:**
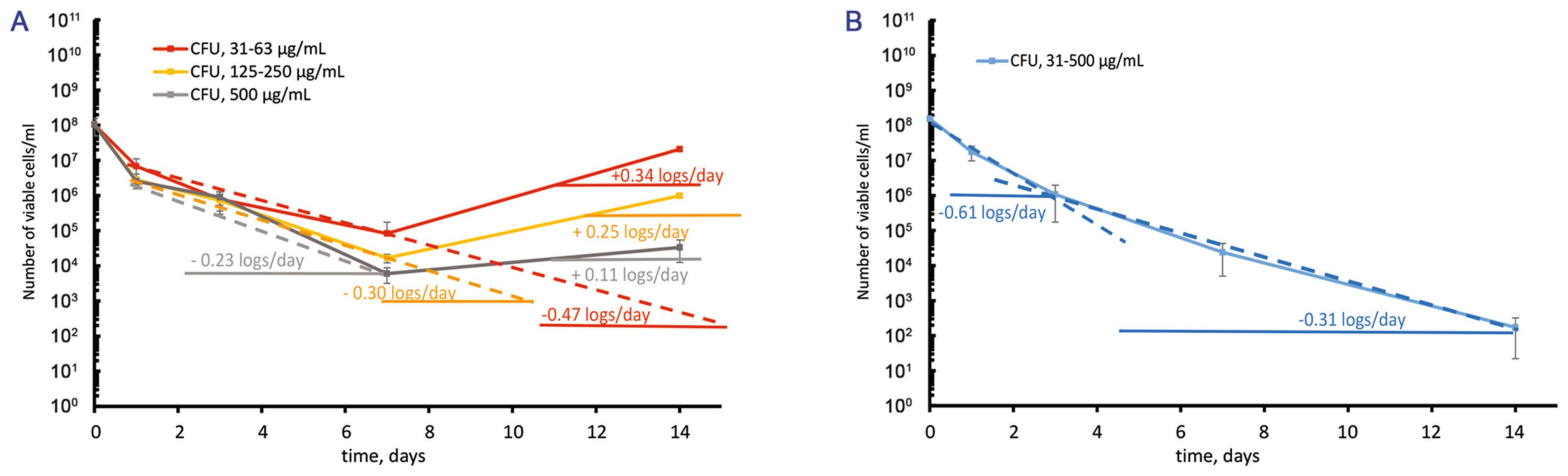
Contrast response of *M. abscessus* cultures grown to the stationary phase to increasing over-MBC moxifloxacin concentrations. The graphs show the corresponding groups with statistically significant differences (*p* < 0.05). **(A,B)** The CFU/mL changes for cultures grown in the complete or potassium-free Sauton media, respectively.

Since CFU-based assays alone may overestimate antibacterial efficacy, as observed with *M. abscessus* (Mulyukin et al., 2023), the most probable number (MPN) test was also used to estimate viable bacteria counts following treatment with moxifloxacin at concentrations well above the MBC. However, both MPN-and CFU-based assays yielded similar numbers of viable cells in both moxifloxacin-free and treated cultures (Supplementary Figures 2B,D, 3B,D), indicating a bactericidal effect rather than the induction of “non-culturability” in the presence of this antibiotic. For pairwise comparisons, we selected groups with statistically significant differences (*p* < 0.05) from the control and other experimental groups. The original data sets (Supplementary Figures 2, 3) were transformed into the plots shown in Figures 2, 3.

### 3.3 Response of mid-log *Mycobacterium abscessus* cells to moxifloxacin

Treatment of suspensions with 5ˑ10^7^ CFU/mL (OD_600_ = 0.1) from actively growing cultures in complete Sauton medium with increasing over-MBC moxifloxacin concentrations caused a profound killing effect (1.13 ± 0.01, 1.34 ± 0.02, and 1.40 ± 0.01 log_10_ units/days for concentrations 31–62, 125–250, and 500 μg/mL, respectively) within 3 days (Figure 2A). The subsequent regrowth of surviving subpopulations was dependent upon the concentration of moxifloxacin. As for treatments with 1–2 MBCs (31 and 62 μg/mL), the regrowth started from day 3 with the rate of 0.50 ± 0.01 log_10_ units/day, yielding the cell numbers by day 14 as in the control antibiotic-free cultures (Figure 2A). The response to exposure to 4–8 MICs of moxifloxacin involved an intermediate stage (days 3 to 7) when insensitive subpopulations were killed at a lower rate (0.18 ± 0.01 log_10_ units/day) than the susceptible subpopulation (1.34 log_10_ units). The number of viable cells declined insignificantly, within one order of magnitude (Figure 2A). This intermediate stage was followed by slow regrowth (at a rate of 0.29 ± 0.01 log_10_ units/day) as in the case of treatments with 4–8 MBCs (125–250 μg/mL). The highest moxifloxacin concentration (16 MBCs, 500 μg/mL) prevented the regrowth (Figure 2A). In addition, we performed the following assay. Colonies on agar medium upon plating of survived cells (7 days after treatment with 8 MBCs of moxifloxacin) were inoculated to fresh Sauton broth, grown to the mid-log phase, and subjected to the same antibiotic treatment according to the guides (Balaban et al., 2019). CFU assays showed no substantial increase in the size of survivors after the second round of exposure to moxifloxacin.

*Mycobacterium abscessus* cells that were grown to the mid-log phase in a potassium-free Sauton medium displayed a similar response upon exposure to moxifloxacin as in the case of the complete medium (Figure 2B). The major statistically significant difference (*p* < 0.05) in the behavior of survived subpopulations in these media was the faster regrowth (0.49 ± 0.01 log_10_ units/day) starting from day 7 and yielding the higher numbers of cells (10^7^ CFU/mL, Figure 2B) as with K^+^ depletion than for cells in the complete medium (regrowth at 0.29 logs/day to 10^5^ CFU/mL, days 7 to 14, Figure 2A) for the exposures to 4–8 MBCs of moxifloxacin. The highest moxifloxacin dose (16 MBCs, 500 μg/mL) was sufficient to cause severe killing of an antibiotic-insensitive subpopulation pre-formed in K^+^ free medium to a residual level of 10^2^ CFU/mL by day 14 (Figure 2B).

### 3.4 Response of stationary-phase *Mycobacterium abscessus* cells to moxifloxacin

Before treatment with moxifloxacin, stationary-phase *M. abscessus* cultures (12–14 days) were diluted to OD_600_ = 0.1 with the spent medium in which cells grew. After the challenge of stationary cells in the complete Sauton medium to moxifloxacin 1–8 MBCs, we observed a gradual decrease (0.23 ± 0.01–0.47 ± 0.01 log_10_ units/day) in the viable cell numbers during 7 days and then regrowth (Figure 3A). A stable stage with unchanged numbers of viable cells (1–3·10^4^ CFU/mL) was observed for stationary-phase cultures grown in the complete medium and exposed to 500 μg/mL of moxifloxacin (Figure 3A).

In contrast, antibiotic-insensitive subpopulations in stationary-phase *M. abscessus* cultures under potassium deficiency experienced gradual killing (0.31 ± 0.01–0.61 ± 0.01 log_10_ units/day) during exposure to moxifloxacin across a wide concentration range (1–16 MBCs) with no subsequent regrowth (Figure 3B). By day 14, the residual number of viable cells was approximately 1·10^2^ CFU/mL (Figure 3B). Thus, the behavior of a pre-formed fraction of survivors in stationary-phase cultures exposed to moxifloxacin is dependent on the availability of potassium ions in the growth medium.

### 3.5 Enumeration of antibiotic-insensitive surviving fractions

To enumerate *M. abscessus* survivors that remained viable during exposure to moxifloxacin, we followed established guidelines for measuring persisters, defined as a small subpopulation of bacteria that are killed by antibiotics at a slower rate than the susceptible population. The size of this subpopulation shows a weak dependence on concentration, resulting in a biphasic killing curve (Balaban et al., 2019).

Both killing curves, “CFU–time” for varying concentrations of moxifloxacin (Supplementary Figures 2, 3) and “CFU–concentration” at a selected time point after antibiotic treatment, were analyzed (Supplementary Figure 4). As the elimination of moxifloxacin-sensitive cells occurred within 3 days of exposure, dose-killing assay results were considered for this time frame.

**Figure 4:**
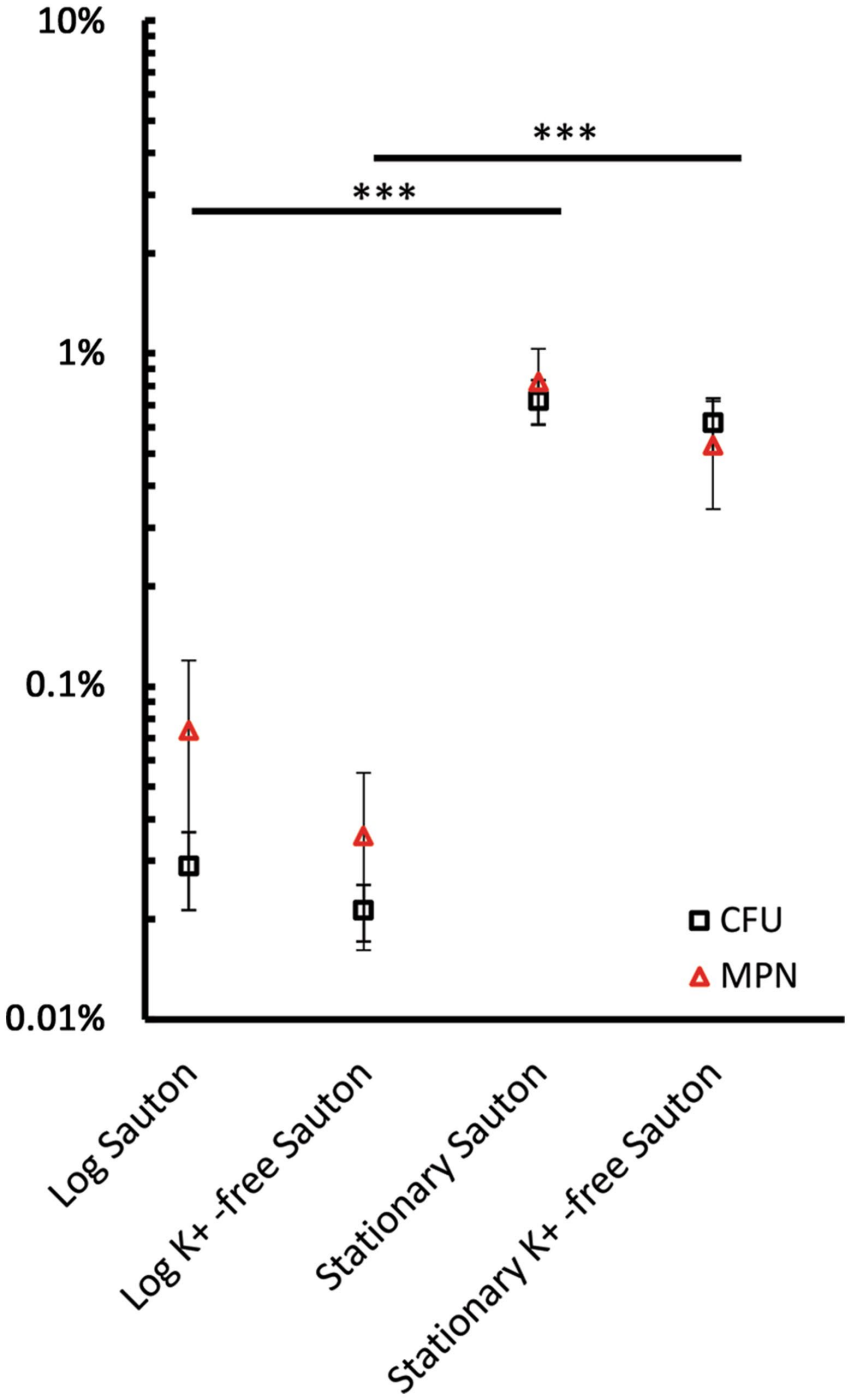
The percentage of survivors in mid-logarithmic and stationary-phase *M. abscessus* cultures grown in complete and potassium-free Sauton medium was estimated upon counting viable cells using CFU and MPN assays. Cultures were exposed to 2–16 MBCs of moxifloxacin and incubated for 3 days. Dose-kill curves for this time point are shown in Supplementary Figure 4. The percentage of survivors was determined from the residual CFU/mL and MPN/mL numbers normalized for the numbers of viable cells prior to exposure to moxifloxacin. Estimations of survivor fractions from CFU and MPN counts are marked here with squares and triangles. The experiments were performed independently twice in triplicates; the mean values and standard deviations are shown here: ***p < 0.005, unpaired *t*-test. Note the dynamic changes in the numbers of drug-tolerant survivors during prolonged incubation, as illustrated in Figures 2, 3.

As for mid-logarithmic and stationary-phase *M. abscessus* cultures in complete Sauton medium, the killing effect was not dependent substantially upon the concentration of the antibiotic starting from 63 to 500 μg/mL as the CFU numbers reached a plateau or changed insignificantly (Supplementary Figure 4). According to the mean viable cell numbers at the plateau level, the percentage of survivors was estimated as being 0.03% (1.2·10^4^ CFU/mL) and 0.72% (7.5·10^5^ CFU/mL) of the CFU before the antibiotic treatment for mid-log and stationary cultures, respectively (Figure 4). The fractions of survivors that remained viable after exposure to moxifloxacin constituted 0.02% (8.1·10^3^ CFU/mL) and 0.62% (9.8·10^5^ CFU/mL), respectively, for mid-log and stationary cultures grown in K^+^-free medium, respectively, as with the complete Sauton medium (Figure 4).

The size of a relatively stable surviving fraction was determined from time-killing curves for the highest moxifloxacin concentration (16 MBCs, 500 μg/mL) at which regrowth did not occur in all antibiotic-exposed groups (Figures 2, 3) and for the end of monitoring (day 14). A fraction of survived cells in the stationary-phase cultures in a complete Sauton medium accounted for 0.03% (~2·10^4^ CFU/mL) (Figure 3A). As with the rest of the moxifloxacin-exposed cultures, the size of a stable surviving fraction was 10^2^ CFU/mL (Figures 2, 3B).

MPN assay was useful in improving the counting of viable cells after moxifloxacin removal rather than estimating the percentage of survivors. Thus, the numbers of viable surviving cells from MPN assays ranged from 10^5^ to 10^6^ cells/mL (vs. 10^4^ CFU/mL) for moxifloxacin-challenged mid-logarithmic cultures (Supplementary Figures 4A,B). The MPN numbers of survivors in stationary-phase cultures, which withstood the antibiotic attack, were tenfold higher than CFU numbers (Supplementary Figures 4C,D).

### 3.6 Changes in the cellular integrity and morphology after short-term exposure to moxifloxacin in high concentrations

The slow and incomplete killing of *M. abscessus* during a 24-h incubation with moxifloxacin (Figures 2, 3) suggests significant heterogeneity within the antibiotic-exposed populations in terms of structural organization, indicating the presence of different cell types. To further investigate cell morphology and integrity, we conducted electron microscopy examinations on untreated specimens that were only washed as well as on thin sections prepared using the standard sample preparation protocol involving fixation and chemical treatments.

**Figure 5:**
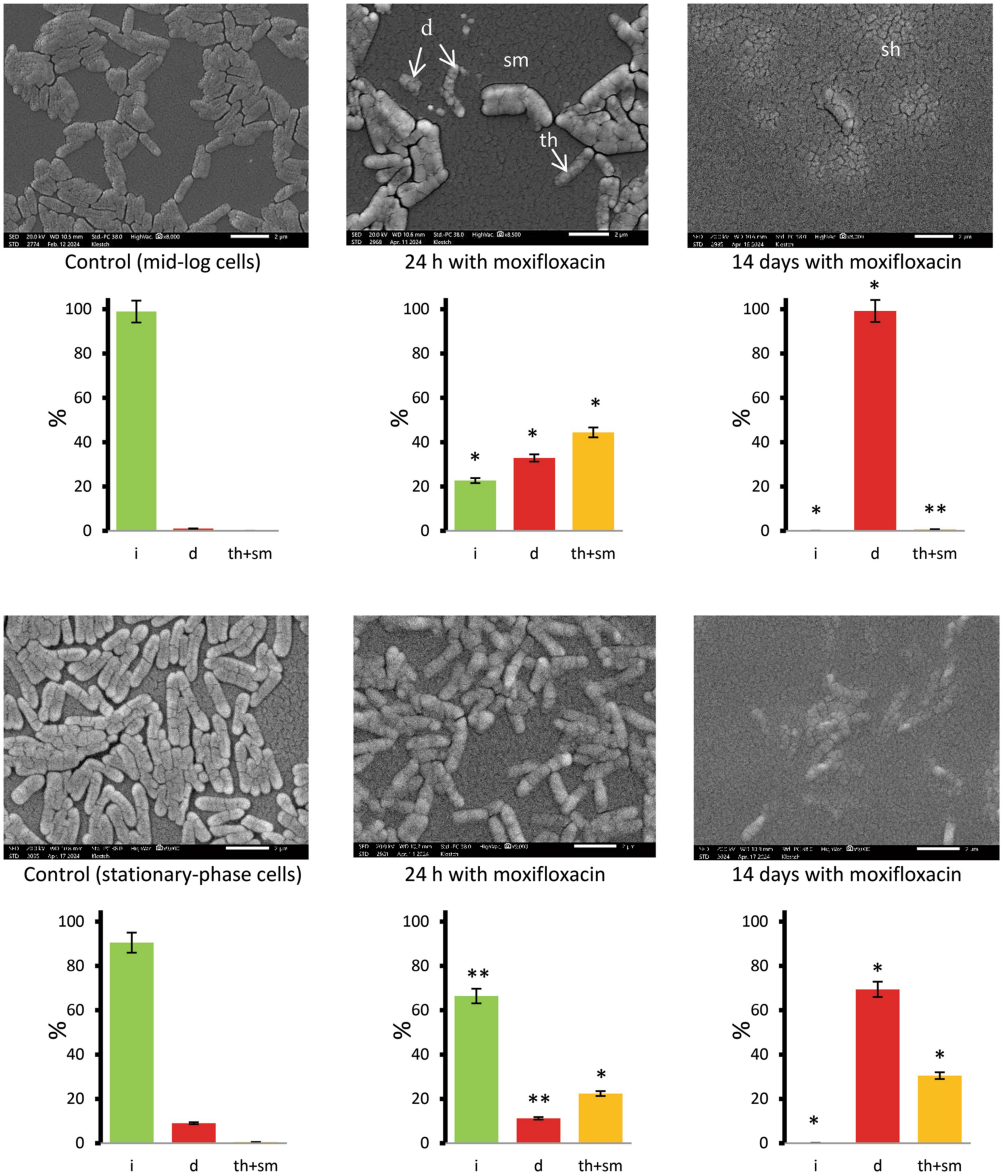
SEM images of *M. abscessus* cells in mid-logarithmic (upper row) and stationary-phase (bottom row) cultures grown in complete Sauton medium in the control and after exposure to moxifloxacin (500 μg mL^−1^) and incubation for 24 h and 14 days. Diagrams show the percentage of cells with intact (i), destructed (d), and thinned and smooth (th + sm) surfaces based on examinations of 134 to 404 cells for each set of images. Asterisks in plots for experimental groups indicate statistically significant differences (*p < 0.05, **p < 0.01) from the control group. Designations: d, destructed cells; sm, cells with smoothened surface; th, thinned surface; sh, cell shadows. Bars, 2 μm.

SEM examinations of unfixed and washed samples showed signs of destruction among singular or paired cells after 24-h exposure of logarithmically growing cells to the antibiotic (32.9% destructed, statistically different from the control), whereas the surface of clumped cells was intact or showed smoothing and thinning (67.1% combined), with their percentage also being statistically different from the control group (Figure 5, images and diagrams). Stationary-phase cells were less susceptible than mid-log cells to short-term exposure to moxifloxacin; cells with intact, destructed, and thinned-smoothened surfaces accounted for 66.4, 11.2, and 22.4%, respectively (Figure 5), compared to the corresponding proportions in antibiotic-challenged mid-log cultures.

TEM with EDX spectroscopy-based analysis of the same samples demonstrated changes in K^+^ levels in mycobacteria treated with high doses of moxifloxacin (16 MBC) (Supplementary Figure 5 and Supplementary Table 2). Based on K^+^ mapping for individual cells in four randomly selected fields (Supplementary Figure 6), we could estimate the total percentage of K^+^-lacked cells in 24-h moxifloxacin-exposed mid-log or stationary-phase as 43.7 ± 4.3%. It is important that aggregated cells in treated cultures contained K^+^-retaining (87.6%) and K^+^-free cells (12.4%), whereas 91.2% of single cells lost K^+^ and the remaining 8.8% showed a detectable potassium level (Supplementary Figure 6). This difference in the distribution of K^+^-containing cells between aggregates (clumps) and singular cells was statistically different (*p* < 0.05). In some intact cells, there were areas of non-uniform phosphorus distribution (Supplementary Figure 5).

**Figure 6:**
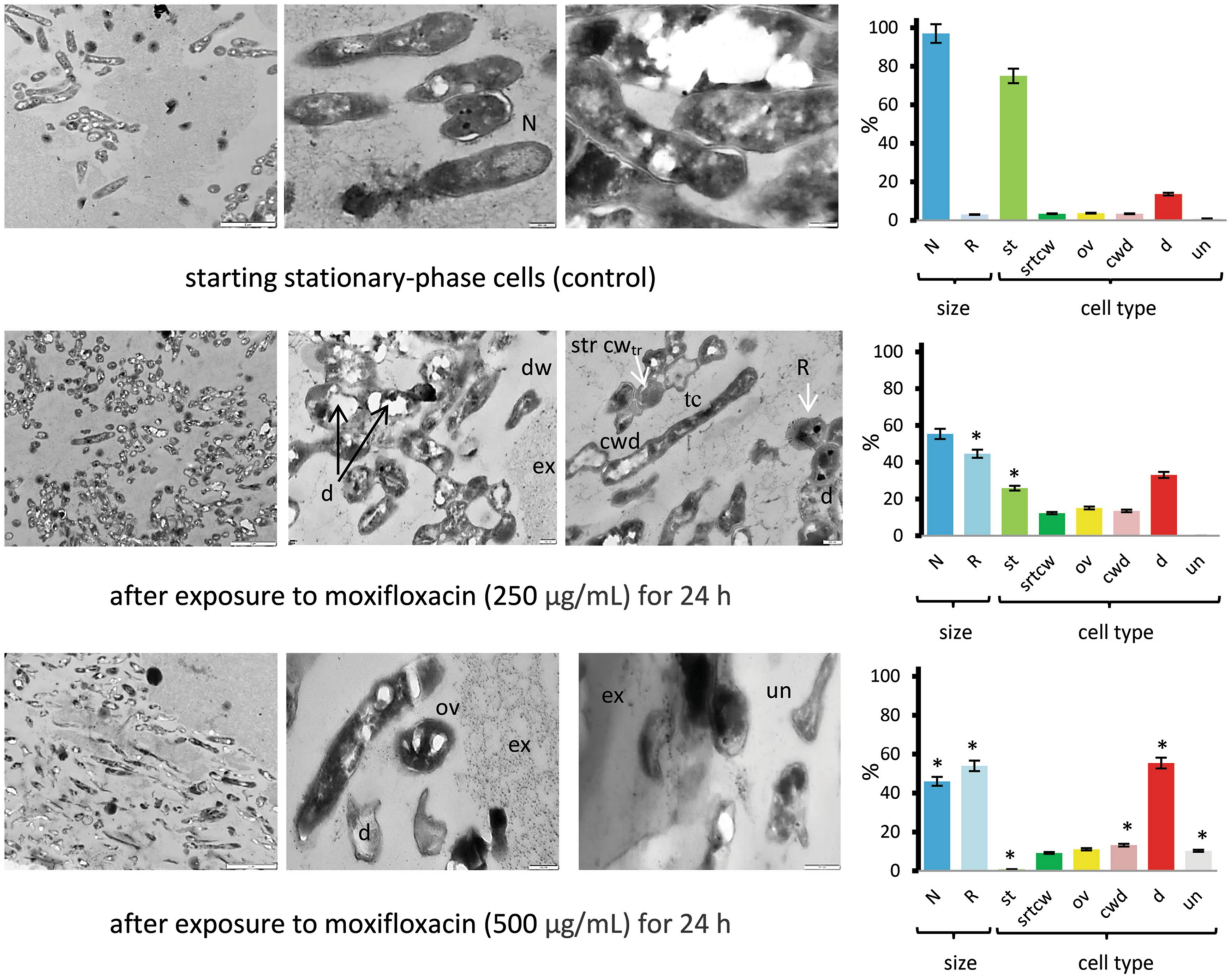
TEM images of thin sections for the control and moxifloxacin-treated *M. abscessus* cultures. Cells were grown in Sauton medium broth to the stationary phase and incubated with the antibiotic (250 or 500 μg mL^−1^) for 24 h. Diagrams show the percentage of cells with more than twice the reduced length and width (R) than 1.70 ± 0.36 μm × 0.34 ± 0.07 μm (L × W) for stationary cells and the proportions of the above-designated cell types. The data were obtained by counting 106 to 379 cells in each set of images. Asterisks in plots for experimental groups indicate statistically significant differences (*p < 0.05) from the control group. Designations: N, cells with size and morphology typical of stationary-phase cultures; R, reduced size; cwd, cell-wall-deficient cells; d; destructed cells; dw, dwarf cells; ex, extracellular material; str cw_tr_, stratified cell wall (transverse sections); ov, ovoid cells; th, thin cells; st, cells with morphology as in the stationary-phase cultures; un, unusual morphological type. Bars: 2 μm (left column images); 200 nm (the middle and right images). Enlarged images are shown in Supplementary Figure 7.

Thin-sectioning TEM proved the presence of both dead mycobacteria with disrupted cell walls and signs of lysis and morphologically altered cells in post-stationary-phase *M. abscessus* populations exposed to moxifloxacin (500 μg/mL, 24 h), which were distinct from cells in the control stationary-phase cultures (Figure 6 and Supplementary Figure 7). The presence (55.4%) of TEM images of destructed cells (a category d) and the high proportion (54%) of the cells with reduced size on the longitudinal and/or transverse sections (a category R) were statistically significantly different (*p* < 0.05) from the control non-treated bacteria (Figure 6). In the case of stationary-phase cultures exposed to 250 μg/mL of moxifloxacin for 24 h, different cell types were observed on TEM images (Figure 6 and Supplementary Figure 7). The significant difference from the control group was the relative abundance of cells (44.6%) with the reduced size (Figure 6, diagrams). Some bacteria in this group (250 μg/mL of moxifloxacin, 24 h) exhibited on the transverse sections a dense and stratified cell wall (strcw category) with an expanded electron-transparent layer between the outer mycolic acid layer and cytoplasmic membrane. Intact cells were attached to dead or destructed neighbors or embedded in vast extracellular material visible as netlike structures to be formed from released polymers or debris (Figure 6).

### 3.7 Morphology of cells in populations after prolonged exposure of stationary-phase cultures to moxifloxacin in the highest concentrations

SEM and TEM-EDX spectroscopy with mapping of carbon, phosphorus, sulfur, potassium, and other chemical elements proved the biocidal effect of moxifloxacin. SEM enabled the visualization of predominant ‘cells’ with the destructed (d category) or thinned and smoothened (th + sm category) surface. Cells with intact surfaces were not detected in stationary-phase culture on the complete Sauton medium after long exposure (14 days) to the highest moxifloxacin concentration (16 MBCs) (Figure 5, right images) upon examining more than twenty SEM fields.

TEM-EDX spectroscopy showed that cell remnants lost total carbon, oxygen, phosphorus, and potassium, as it is seen on colored images (Supplementary Figure 5) or judged from the relative contents tabulated for an entire examined field with ‘cells’ and surroundings (Supplementary Table 2). The loss of these elements in individual cells can be explained by the removal of cellular components from lysed or destructed cells present in the antibiotic-challenged culture after washing biomass with water, whereas intact cells retain the intracellular constituents. A uniform distribution of total carbon on elemental maps without appearance in cell remnants (Supplementary Figure 5, the left image in the bottom row) and reliably detectable C levels in the integral spectra for fields (Supplementary Table 2) can be due to the presence of extracellular material that was not removed after washing used for sample preparation. However, SEM and TEM-EDX spectroscopy detected no low-abundant intact cells in such small samples (5–10 μL), and concentrating procedures with additional washing steps and supernatant removal were undesirable due to possible losses of biomaterial.

**Figure 7:**
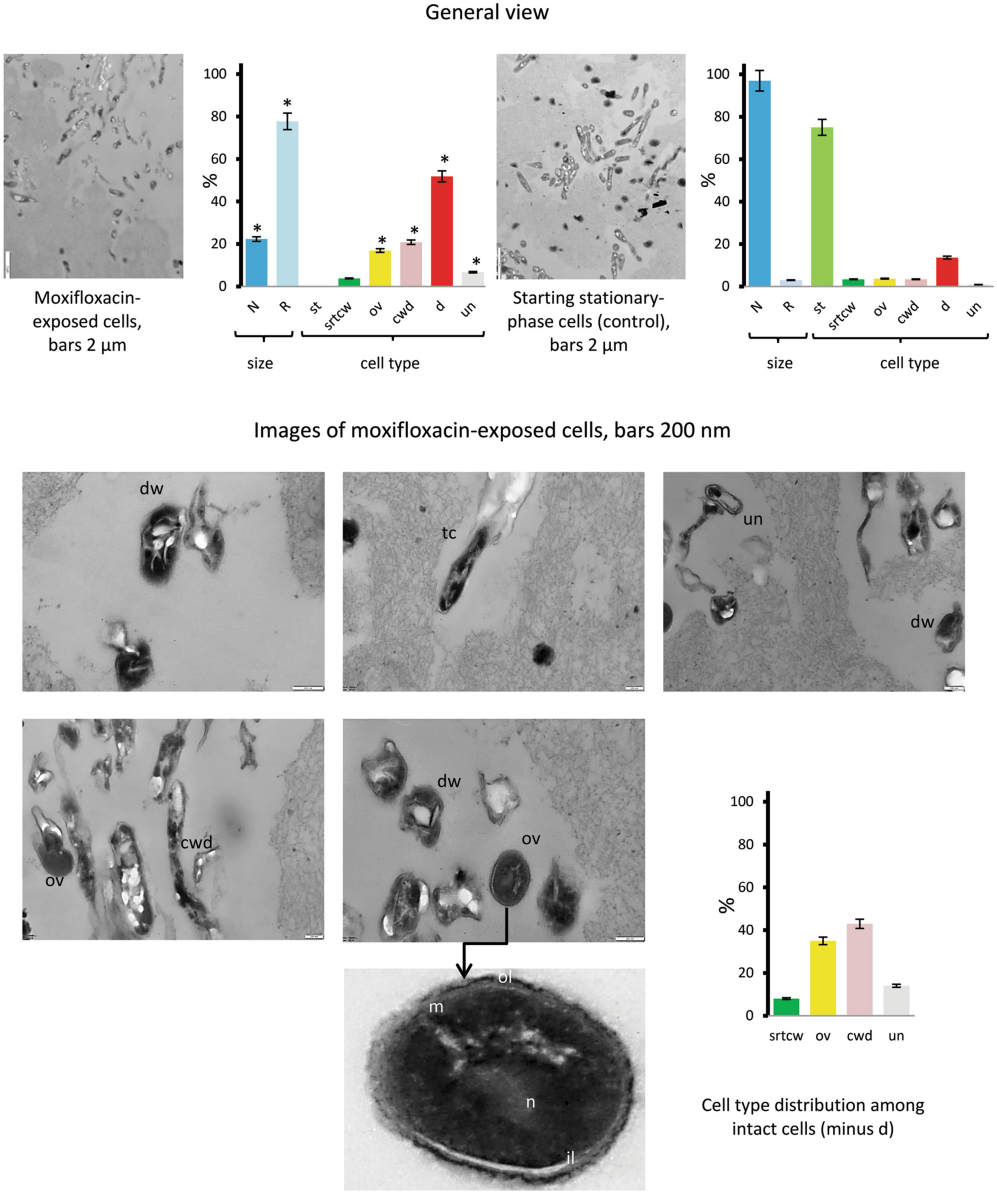
Thin sections of untreated stationary-phase *M. abscessus* cells and cells after prolonged incubation with moxifloxacin (500 μg/mL, 14 days) as viewed under a transmission electron microscope. Diagrams show the percentage of cells with more than twice the reduced length and width (R) than 1.70 ± 0.36 μm × 0.34 ± 0.07 μm (L × W) for stationary cells and the proportions of the above-designated cell types. The data were obtained upon examinations of 106 cells. Asterisks in plots indicate statistically significant differences (*p < 0.05) from the control group. Designations: N, cell with size and morphology as in the stationary-phase cultures; R, reduced size; cwd, cell-wall-deficient cells; d; destructed cells; dw, dwarf cells; ex, extracellular material; ol, outer mycolic acid layer; il; individual layers in cell wall; m, cytoplasmic membrane; n nucleoid; st, cells with morphology as in the stationary-phase cultures; str cw_tr_, stratified cell wall (transverse sections); ov, ovoid cells; th, thin cells; un, unusual morphological type. Bars: 2 μm (images in the left row); 200 nm (the middle and right images). Enlarged images are shown in Supplementary Figure 7.

TEM examinations of thin sections from concentrated cultures exposed to the highest concentration of moxifloxacin for 14 days revealed a diversity of morphological types, including (i) dwarf cells (dw) with irregular shapes (6.7% of total intact cells in the category with ‘unusual’ morphology), (ii) thinned, electron-dense, cell-wall-deficient (cwd) cells (20.8%), (iii) ovoid (ov) cells (16.9%), and cells with distinct morphological features, such as stratified cell walls, compact nucleoids, and finely grained cytoplasm (Figure 7, images and diagrams). Additionally, we included tailed cells with thin envelopes and condensed protoplasts or bodies released upon cell wall destruction in the cwd category. The proportions of morphological types showing significant differences (*p* < 0.05) compared to the control group are presented in Figure 7. Notably, cells resembling stationary-phase cells (st category) were absent. Cells with reduced size accounted for 77.7% of the total, a significantly higher percentage (*p* < 0.05) than in the control group (3%).

## 4 Discussion

### 4.1 Different behavior of *Mycobacterium abscessus* populations under exposure to moxifloxacin

This study’s novel and major result is that the fate of *in vitro*-cultured *M. abscessus* populations under prolonged exposure to moxifloxacin *in vitro* is pre-determined by cultivation history. As for mid-log *M. abscessus* cultures, the impact of potassium deficiency on prolonged moxifloxacin activity was not substantial, except for the efficient regrowth observed for populations grown in K^+^-depleted medium and then treated with 4–8 MBCs of the antibiotic (Figure 2B) as compared to cultures grown within the balanced Sauton medium (Figure 2A). The effect of the medium used was impressive for stationary-phase cultures as judged from distinct responses, depending on moxifloxacin concentrations, for cells grown in the complete medium (Figure 3A) and gradual diminution of an insensitive subpopulation upon exposure to all antibiotic doses (1–16 MBCs) as for K^+^ depletion (Figure 3B). So, the imposed potassium deficiency appeared to contribute to the locking of a fraction in stationary-phase culture to be recalcitrant to moxifloxacin in a state preventing the regrowth (Figure 3B). Remarkably, the lack of K^+^ in the medium prevented further rebound growth after prolonged exposure to stationary-phase *M. abscessus* (Figure 3B) but not mid-log cells (Figure 2B).

It is not simple now to explain why potassium depletion was critical for stationary-phase cells of *M. abscessus*. Generally, external potassium concentrations are known to influence gene expression, antimicrobial resistance, and biofilm formation in various bacterial pathogens, and potassium transport systems are crucial to fulfill nutritional and chemiosmotic requirements (see the review by Do and Gries, 2021, and references therein). Potassium depletion is a severe stress factor since K^+^ is known to be crucial for maintaining an electrochemical gradient and a proton-motive force, regulating intracellular pH and osmotic pressure in mycobacterial cells (Epstein, 2003; Castañeda-García et al., 2011). Potassium deficiency caused an imbalance of energy metabolism in *Mycobacterium tuberculosis*, as demonstrated by transcriptomic and proteomic studies (Salina et al., 2014b). The role of potassium availability in differential gene expression in active and stationary phase cells with or without K^+^-depletion stress remains unknown for *M. abscessus* and needs to be explored. The response of stationary-phase *M. abscessus* cultures (12–14 days) in K^+^-free medium to varying concentrations of moxifloxacin (Figure 3B) was different from the behavior of moxifloxacin-challenged cells grown under the same potassium sequestration condition but stored for 24 and 44 days, and for the aged cultures, the decline in the number of viable survivor cells (especially MPN counts of 10^4^–10^5^ cells/mL) after 14 days of exposure to 3 MBC of moxifloxacin (Mulyukin et al., 2023) was higher than in the case of stationary cultures (10^2^ cell/mL) (Supplementary Figure 3D). It is likely that not so much stationary-phase cultures as aging post-stationary populations are reservoirs for subpopulations that can survive better in the presence of moxifloxacin. As for aging post-stationary cultures, the deepening of dormancy can be an important factor in survival in the presence of antibiotics, as discussed (Ayrapetyan et al., 2018).

### 4.2 Relation to assays of *Mycobacterium abscessus* ‘persisters’

The results of this study can inform the selection of experimental conditions, including variables such as media, time points, and antibiotic concentration, suitable for in-depth studies on persisters. *M. abscessus* proved to be a challenging organism for detecting the minor subpopulations responsible for prolonged survival in the presence of moxifloxacin. Conventionally and repeatedly determined MICs of moxifloxacin, which were measured at 2 μg/mL (Supplementary Table 1) and aligned with previously reported values (Ferro et al., 2016), did not provide sufficient guidance for selecting a concentration range that would result in a plateau of viable cell numbers after antibiotic treatment.

While several MICs are typically sufficient for persister enumeration in other bacteria, such as *P. aeruginosa* (Möker et al., 2010), they were inadequate for *M. abscessus* in our experiments (Figure 1). In fact, hundreds of MICs were required, as shown in our study (Figures 2, 3), similar to findings in persister assays for *M. tuberculosis* (Quigley and Lewis, 2022). The MBC of moxifloxacin for mycobacterial cultures with 5ˑ10^5^ CFU/mL, as determined in conventional assays, can be inferred from the known MBC/MIC ratios ≤4 for bactericidal antimicrobials (Maurer et al., 2014) and was inapplicable for cultures with high cell density (5ˑ10^7^ CFU/mL) suitable for detecting a low-abundant survivor fraction. Indeed, moxifloxacin concentrations, well higher than the MBC (Figure 4), were necessary to enumerate a fraction of survivors in planktonic aerated cultures, which we chose as targets for the antibiotic attack. As for the nutrient-starved and anaerobic *M. abscessus* cultures or biofilms, which are much less susceptible than planktonic cultures (Yam et al., 2020), special conditions, suitable antibiotic concentrations, and sample preparation can be required to implement the classical assay for persister enumeration. Since the biocidal effect of moxifloxacin was gradually developing and occurred by day 3 (Figures 2, 3), it was important to use this time point to enumerate survivors from dose-killing curves. The larger size of survivors in the stationary phase than in preceding mid-log *M. abscessus* cultures (Figure 4) is not surprising and was quite expected from studies of the persister phenomenon (Keren et al., 2004; Möker et al., 2010; Lewis, 2007).

In our study, CFU counting was supplemented with the MPN assay to create conditions for the resuscitation of cells that had lost colony-forming ability. The use of resuscitation procedures for viable-but-non-cells (VBNC) cells, as reviewed by Oliver (2010), is justified since persistence and VBNC states are considered two common survival strategies for bacteria under adverse environmental conditions (Ayrapetyan et al., 2018). Additionally, as reported for *E. coli*, these states may represent either the same dormant phenotype (Kim et al., 2018) or different stages of a single dormancy program (Dewachter et al., 2021).

Drug resistance probably contributes to the recovery of moxifloxacin-insensitive *M. abscessus* sub-populations after both short or prolonged exposure (Figures 2, 3). For an *M. abscessus* subpopulation in mid-log culture exposed to moxifloxacin (8 MBCs, 7 days) and at an intermediate “no-growth” stage with only slight changes in CFU (Figure 2A), the contribution of persisters to the pool of survivors is likely, as indicated by assays showing regrowth of surviving cells after antibiotic withdrawal under the same cultivation conditions and re-treatment with the antibiotic (subsection 3.1).

It should be pointed out that genetically resistant mutants capable of growing in the presence of moxifloxacin have been shown to emerge from persister subpopulations in other mycobacteria (Sebastian et al., 2017; Swaminath et al., 2020). Considering the varied development of small moxifloxacin-insensitive *M. abscessus* subpopulations that either withstand, survive, or perish under specific conditions (Figures 2, 3), the coexistence of genetically based resistance and persistence as a non-genetic form of antibiotic tolerance—viewed as complementary bacterial adaptations to antibiotics (Vogwill et al., 2016)—warrants further investigation.

### 4.3 Lessons from electron microscopy

Methodologically, the combined use of various electron microscopy techniques enabled us to examine cell morphology and structure (SEM and thin section TEM, Figures 5–7) and monitor changes in the composition of biogenic elements and cationic homeostasis using non-invasive TEM coupled with EDX spectroscopy and elemental mapping (Supplementary Figures 5–7 and Supplementary Table 2). It may be useful to note that chemical treatment and fixatives must be avoided during sample preparation for EDX analysis, and suspensions with excessively high cell density are unsuitable.

To the best of our knowledge, EDX-based analysis has not been previously applied to study viable mycobacteria, but it holds promise as a valuable approach for future investigations. Notably, the electron microscopy study provided insights into the delayed biocidal effect of moxifloxacin on *M. abscessus*, attributed to the presence of aggregates that, unlike single cells, were destroyed only after prolonged antibiotic exposure, as evidenced by changes in cell morphology (Figures 5–7) and monitoring of K^+^ loss (Supplementary Figure 6). The reduction in K^+^ signals detected via EDX analysis (Supplementary Figure 6) indicates disrupted homeostasis and membrane destruction, consistent with findings from previous studies on dead cells (Mulyukin et al., 2002).

The important result from our electron microscopy study is the demonstration of the remarkable heterogeneity of *M. abscessus* subpopulations surviving under short or prolonged exposure to moxifloxacin. Likely, a subpopulation of survivors in the stationary phase had exploited different mechanisms of morphological transformation, which were not the same as in the case of aging antibiotic-free cultures of *M. abscessus* (Mulyukin et al., 2023). The subpopulation that survived under a cruel moxifloxacin pressure contained various morphological types (Figures 6, 7). Among them, we observed polymorphic forms resembling cell-wall-deficient (CWD) cells, well-known for mycobacteria and found in host organisms, as comprehensively reviewed by Beran et al. (2006). Some of the cells (Supplementary Figure 7) were similar to CWD cells (named L-forms) observed for ethambutol-treated *M. tuberculosis* and are associated with drug tolerance (Slavchev et al., 2016). It is possible that the embedding in the exopolymer matrix (Figure 6) compensated for the lack of destructed cell walls and helped *M. abscessus* cells to survive. Thus, massive EPS ensured long-term survival for up to 12 months of both antibiotic-sensitive and insensitive subpopulations of pseudomonads in the forms of normal cyst-like and cell-wall-deficient cells (Mulyukin et al., 2017). Surviving *M. abscessus* populations contained intact cells with stratified cell walls (Figure 6 and Supplementary Figure 7). Cells with thickened cell walls were found among antibiotic-exposed mycobacteria (Jakkala and Ajitkumar, 2019; Sebastian et al., 2020). Ovoid cells (Figure 7) resembled in morphological traits the ovoid cells of *M. tuberculosis*, which were characterized as dormant forms based on a sum of their properties (Shleeva et al., 2011). Some morphological types of moxifloxacin-challenged *M. abscessus* populations are similar to other bacteria, *S. aureus* (Liu et al., 2024) and *E. coil* (Kim et al., 2018).

It is commonly accepted and convincingly proved that the persister phenomenon is closely associated with dormancy (Niu et al., 2024; Wood et al., 2013; Kim et al., 2018). Also, it is proposed to consider persisters from non-dormancy standpoints (Umetani et al., 2021; Zou et al., 2021). A consensus may be achieved because the development of a ‘persister’ subpopulation under exposure to antibiotics proceeds via diverse transformation mechanisms, as was already observed for antibiotic-surviving *P. aeruginosa* PAO1 (Mulyukin et al., 2015). Therefore, obtaining pure and enriched fractions of survived cells and characterizing them using viability tests, physiological and biochemical methods, and omics approaches is a fascinating and challenging task, although large volumes of cell cultures will unavoidably be required.

### 4.4 Relevance for combatting *Mycobacterium abscessus* infections

Our *in vitro* study demonstrated that moxifloxacin, at a given concentration (e.g., several MICs or MBCs), exerts distinct effects on *M. abscessus* cells depending on their abundance and physiological state (Figures 1–4). Variations in cell density and activity, the stress of potassium deficiency (Do and Gries, 2021), and persisting states (Yam et al., 2020) can occur in microenvironments of infected cells or tissues, influencing the phenotypic properties of *M. abscessus* and ultimately determining the success of antibiotic therapy (Bumann, 2015).

Given that the response to the same antibiotic treatment may vary for mycobacterial cells likely existing in various physiological states and abundances, this could provide an additional explanation for the controversial results of moxifloxacin application against *M. abscessus* observed in both *in vitro* (Choi et al., 2012; Park et al., 2008; Guo et al., 2024; Nie et al., 2014; Daley et al., 2020; Haworth et al., 2017) and *in vivo* studies (Nie et al., 2020; Ferro et al., 2016; Choi et al., 2011).

The observed and substantial decline in the number of viable *M. abscessus* cells *in vitro* under certain conditions (i.e., K^+^ deficiency) following prolonged exposure to moxifloxacin (Figure 3B and Supplementary Figures 3C,D) appears to be promising. However, the requirement for high concentrations remains a limitation for anti-persister therapy. Since prolonged treatment with high doses of conventional antibiotics is likely to be an ineffective therapeutic approach and may contribute to the development of chronic and relapsing infections, a robust anti-persister strategy is crucial for combatting severe human infections.

Such a strategy may involve several approaches: sensitizing persisters before antibiotic treatment, using adjuvants to enhance antibiotic efficacy, directly eradicating persisters with bioactive molecules that bypass conventional antibiotics, or preventing persister formation altogether, as extensively reviewed for various pathogenic bacteria (Zhou et al., 2023; Defraine et al., 2018). Notably, the membrane-active cationic glycopolymer PAAG (Narayanaswamy et al., 2022) and the conjugated oligoelectrolyte COE-PNH_2_ (Zhang et al., 2024) have been shown to efficiently eradicate *M. abscessus* pesisters.

Additionally, the possibility of locking a moxifloxacin-insensitive subpopulation of survivors in a non-regrowth state, as observed in this study (Figures 2, 3 under specific conditions and concentrations), aligns with the proposed alternative anti-persister strategy of inducing deep dormancy (Zhou et al., 2023).

## Data Availability

The raw data supporting the conclusions of this article will be made available by the authors, without undue reservation.
