# Supplementary material for: Fate of in vitro cultured Mycobacterium abscessus populations when exposed to moxifloxacin
Source: Front Microbiol. 2024 Nov 28;15:1494147. doi: 10.3389/fmicb.2024.1494147 (PMC11635960; doi:10.3389/fmicb.2024.1494147)
Supplement: Supplementary file 1 [file Data_Sheet_1.pdf]

Supplementary Material

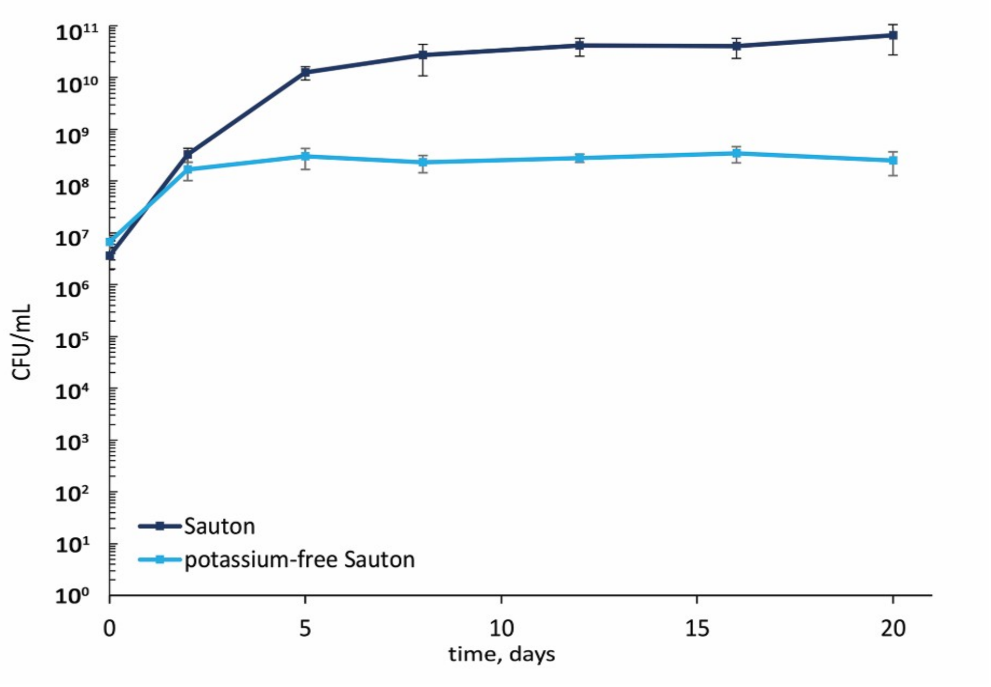

Supplementary Figure 1. *M. abscessus* growth in complete or potassium-free Sauton medium supplemented with 10% ADC and 0.05% Tween-80.

A starter *M. abscessus* mig-log culture was inoculated (0.25%) into Sauton medium, containing: KH_2PO_4, 0.5 g; $MgSO_4{\cdot}7H_2O$, 1.4 g; L-asparagine, 4 g; glycerol, 60 mL; ferric ammonium citrate, 0.05 g; sodium citrate, 2 g; 1% $ZnSO_4 \cdot 7H_2O$, 0.1 mL; H_2O, to 1 L; pH 7.0 (adjusted with 1 M NaOH) or into potassium-free Sauton media with ADC and Tween-80 in which K^+ ions were equimolarly substituted for Na^+ ions (Salina *et al.*, 2014) with addition of 10% ADS and 0.05% Tween-80. Cultures were incubated in loose-capped flasks at 37 °C with shaking at 200 rpm. Culture aliquots were collected at different time points. Tenfold serial dilutions of cultures were plated in triplicates onto 7H10 agar (Himedia, Mumbai, India) plates supplemented with 10% ADC in Petri dishes and incubated at 37 °C for 6 days followed by CFU counting. Experiments were conducted in three biological replications; the mean values and standard deviations are shown.

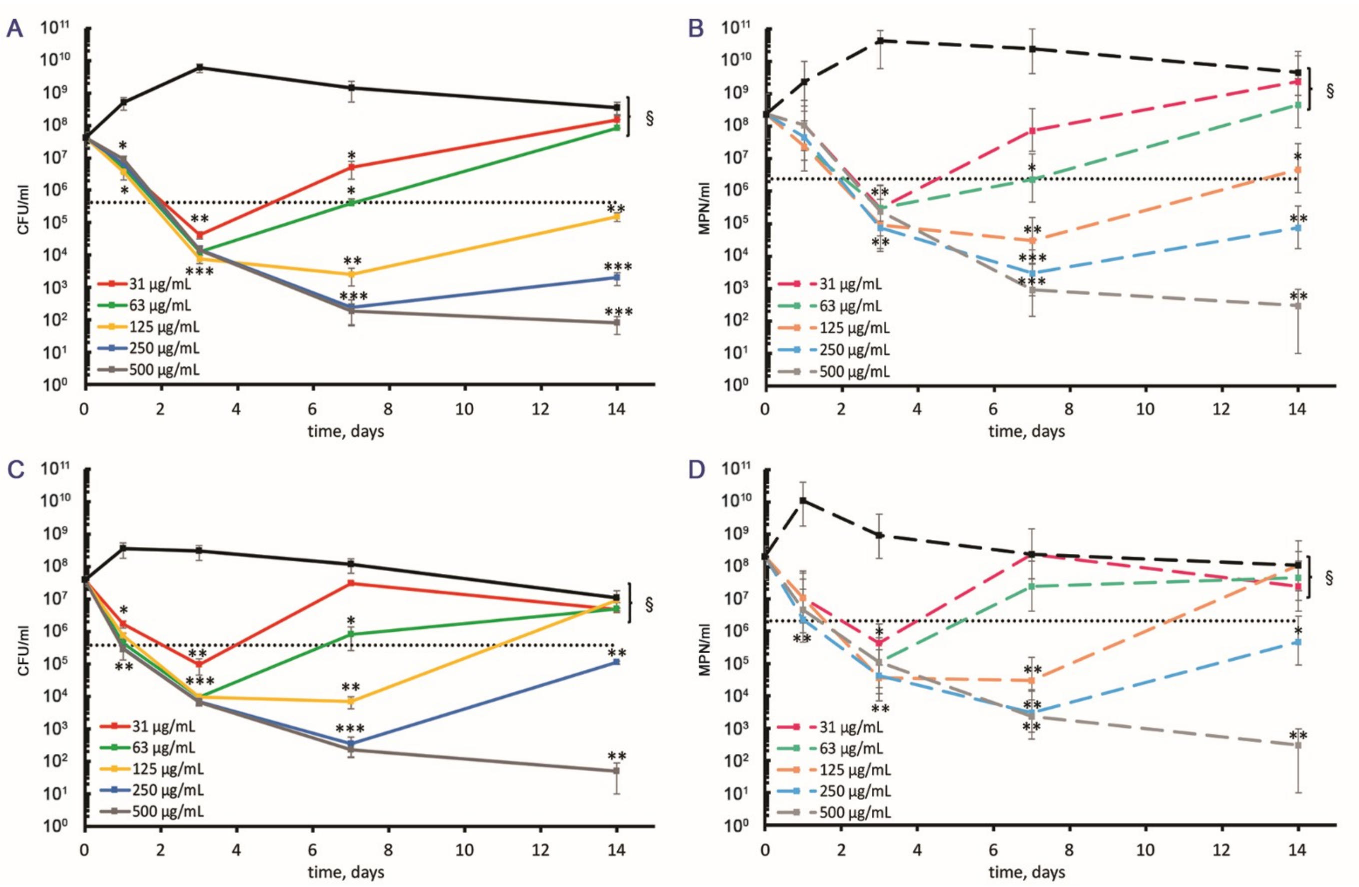

Supplementary figure 2. Response of *M. abscessus* cultures grown to the mid-logarithmic phase in the complete or potassium-free Sauton media and incubated with moxifloxacin added in various concentrations (31 – 500 µg/mL) for up to 14 days. **(A),(C)** Changes in the CFU/ml for cultures grown in the complete or potassium-free Sauton media, respectively. **(B),(D)** Changes in the MPN/ml for cultures grown in the complete or potassium-free Sauton media, respectively.

The experiments were performed two times independently in triplicates; the mean values and standard deviations are shown here; *$p < 0.05$, **$p < 0.01$, ***$p < 0.005$, data were analyzed using Student's unpaired *t*-test and $p < 0.05$ was considered statistically significant in comparison to a non-drug control values. Statistically insignificant differences between experimental groups are marked with §, and these groups were excluded in Figure 2. Dotted lines indicate the minimum threshold for 2-log_{10}-drop of cell viability to monitor time by which mycobactericidal effect was achieved.

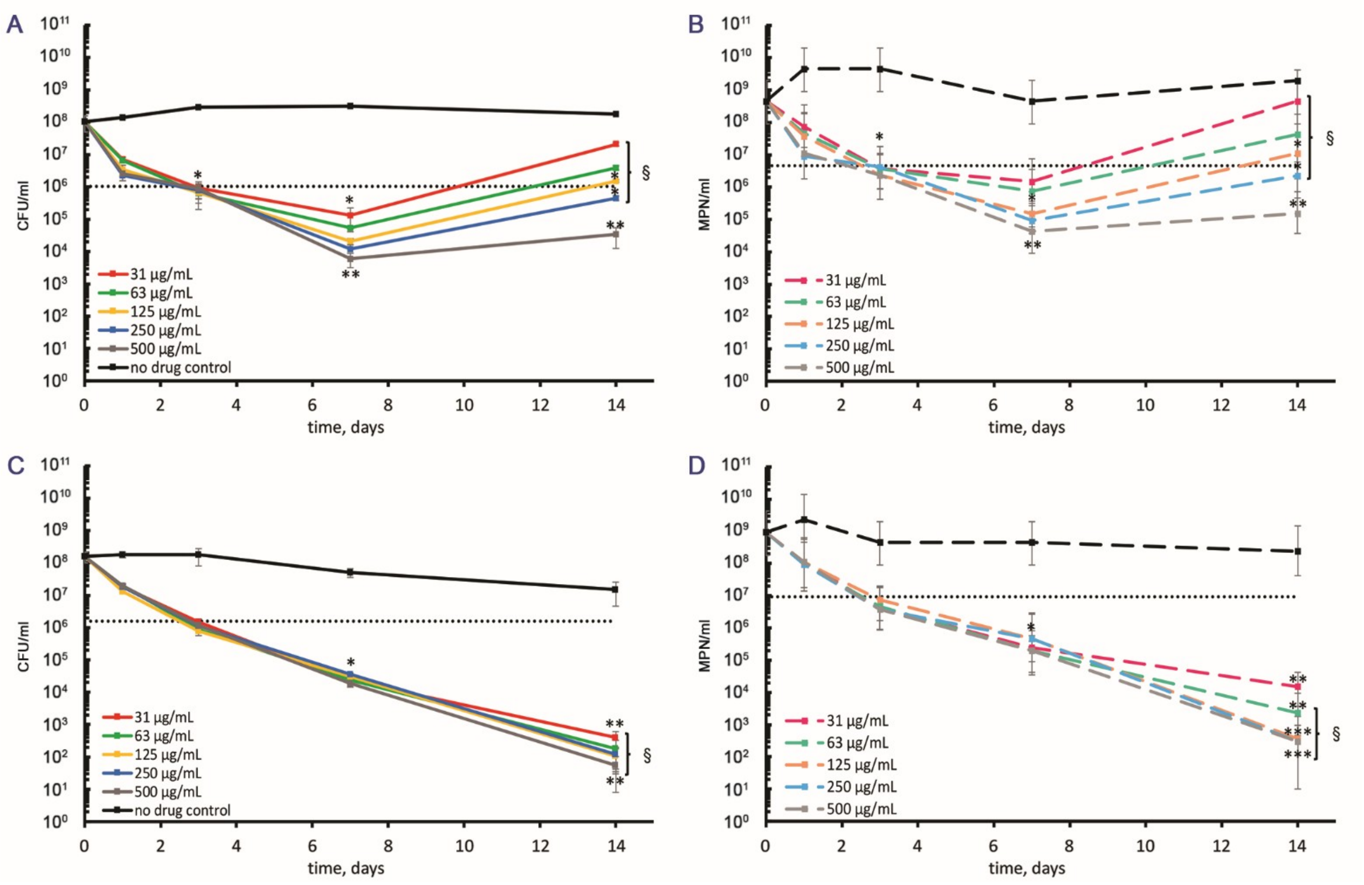

Supplementary figure 3. Contrast response of stationary-phase *M. abscessus* grown in the complete or potassium-free Sauton media to moxifloxacin exposure. **(A),(C)** Changes in the CFU/ml for cultures grown in the complete or potassium-free Sauton media, respectively. **(B)**, **(D)** Changes in the MPN/ml for cultures grown in the complete or potassium-free Sauton media, respectively. The experiments were performed two times independently in triplicates; the mean values and standard deviations are shown here; *$p < 0.05$, **$p < 0.01$, ***$p < 0.005$, data were analyzed using Student's unpaired *t*-test and *$p < 0.05$ was considered statistically significant in comparison to a non-drug control values. Statistically insignificant differences between experimental groups are marked with §, and these groups were excluded in Figure 3. Dotted lines indicate the minimum threshold for 2-$\log_{10}$-drop of cell viability to monitor time by which mycobactericidal effect was achieved.

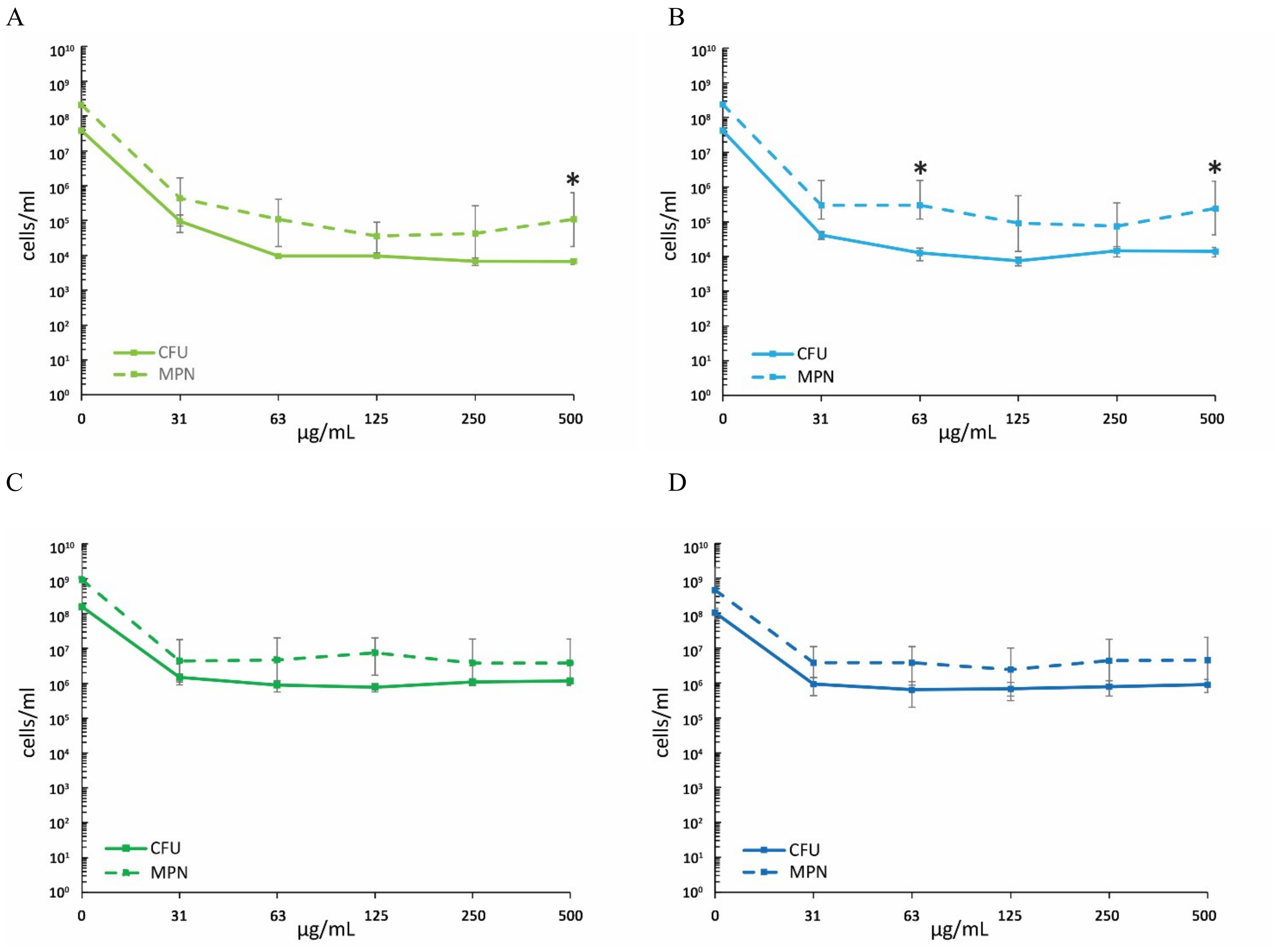

Supplementary Figure 4. Dose-killing curves of *M. abscessus* after 3-day exposure to moxifloxacin (31 – 500 µg/mL). Changes in the CFU and MPN values are shown. **(A)**, **(B)** Mid-logarithmic cultures in complete or potassium-free Sauton media, respectively. **(C)**, **(D)** Stationary cultures in complete or potassium-free Sauton media, respectively. The experiments were performed at least two times independently in triplicates; the mean values and standard deviations are shown here; data were analyzed using Student's unpaired *t*-test and $*p < 0.05$ was considered statistically significant.

A

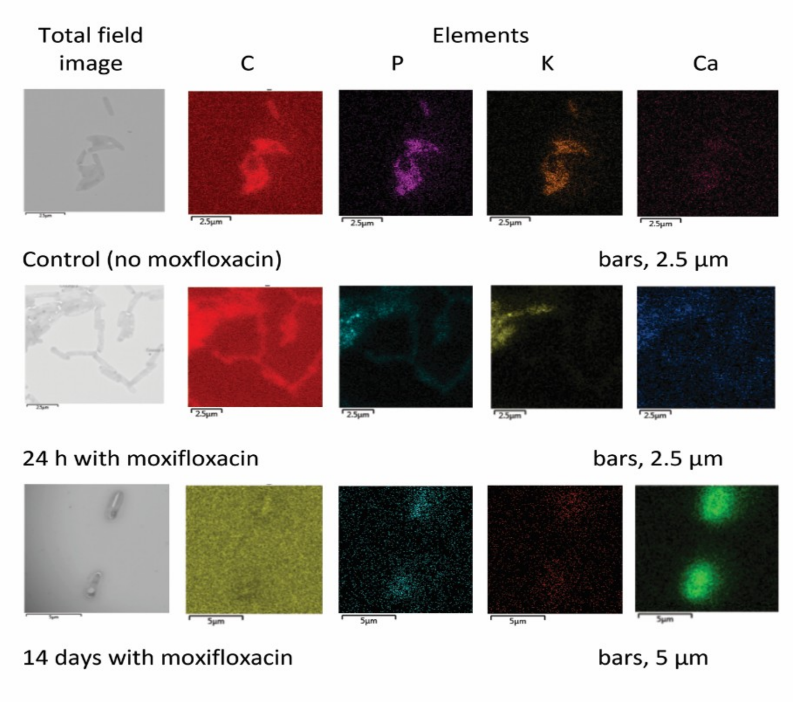

B

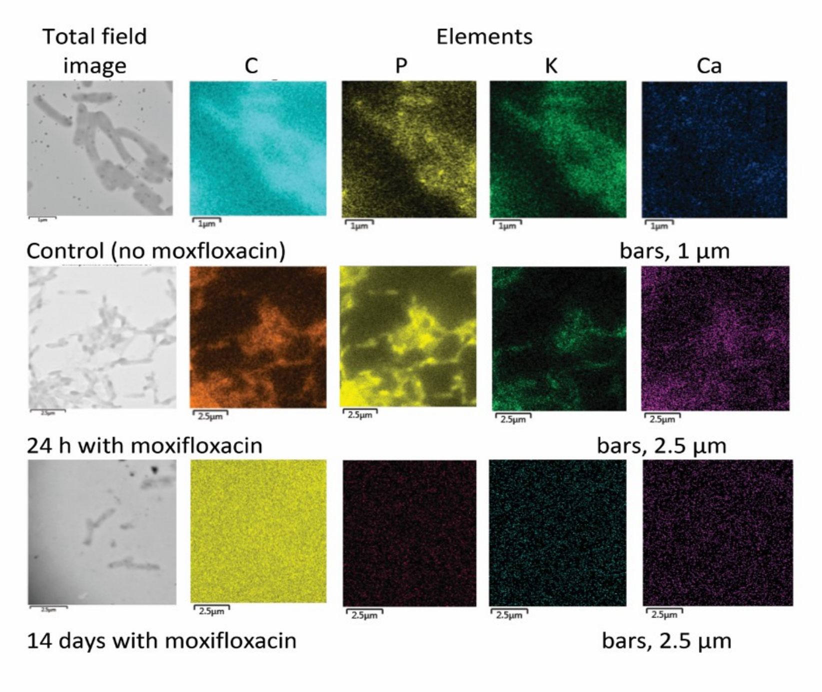

Supplementary Figure 5. TEM-EDX analysis: images of fields with *M. abscessus* cells and maps of selected elements (C, P, K, Ca). SEM-images before and after exposure to moxifloxacin (500 µg/mL) and incubation for 24 h and 14 days. **(A)** Mid-logarithmic cells (complete Sauton medium). **(B)** Stationary-phase cells (complete Sauton medium).

A

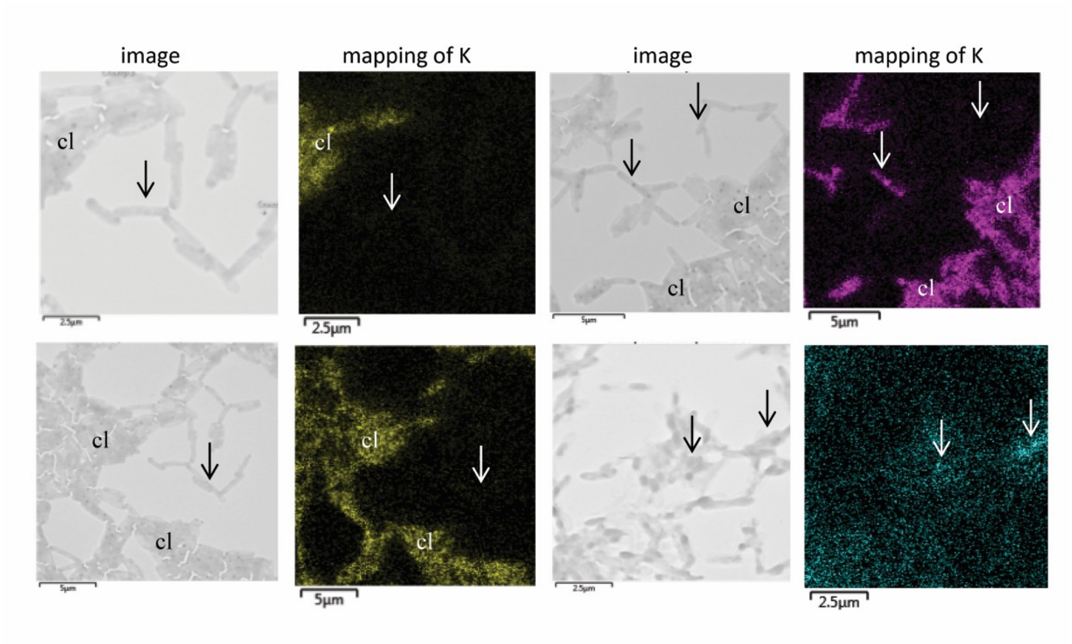

B

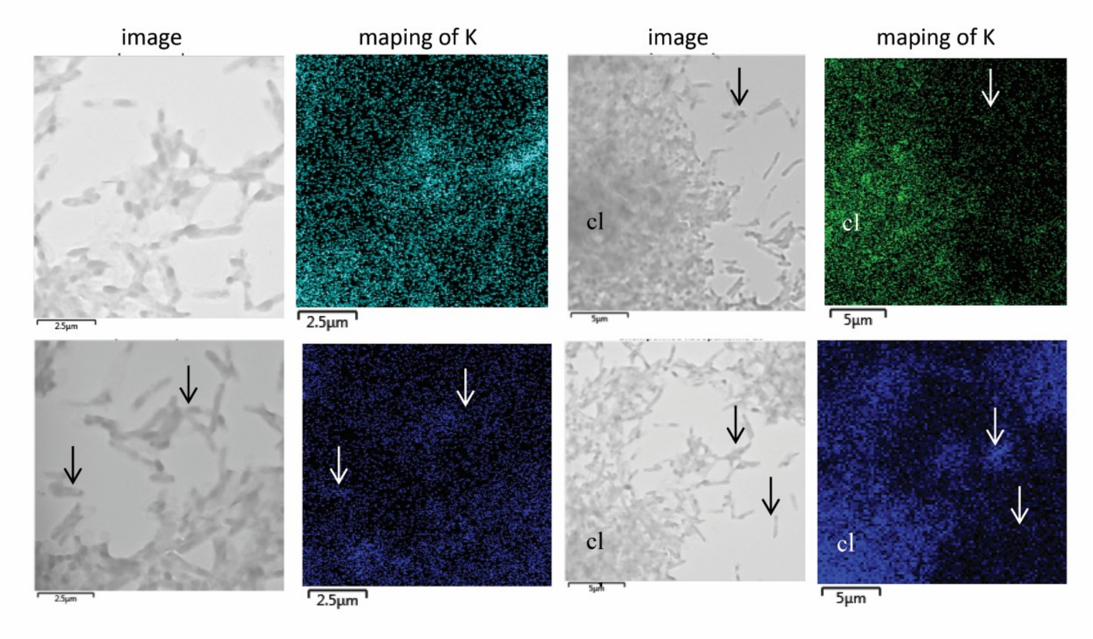

Supplementary figure 6. TEM and EDX analysis. Pairwise comparisons of total images and their maps for potassium show a leakage of K^+ from singular *M. abscessus* cells after exposure to moxifloxacin (500 µg/ml) for 24 h. Cells in clumps retained a residual intracellular potassium level and would undergo further destruction. **(A)** Mid-logarithmic cultures. **(B)** Stationary-phase cultures. Designations: cl, cell clumps; some singular cells are marked with arrows. Cells with lost K+ comprised totally 43.7% ±4.3**%.** Clumps: 87.6% ± 11.4% cells with detectable K^+. Single cells: 8.8% ± 5.4% with detectable K^+ (p< 0.05)

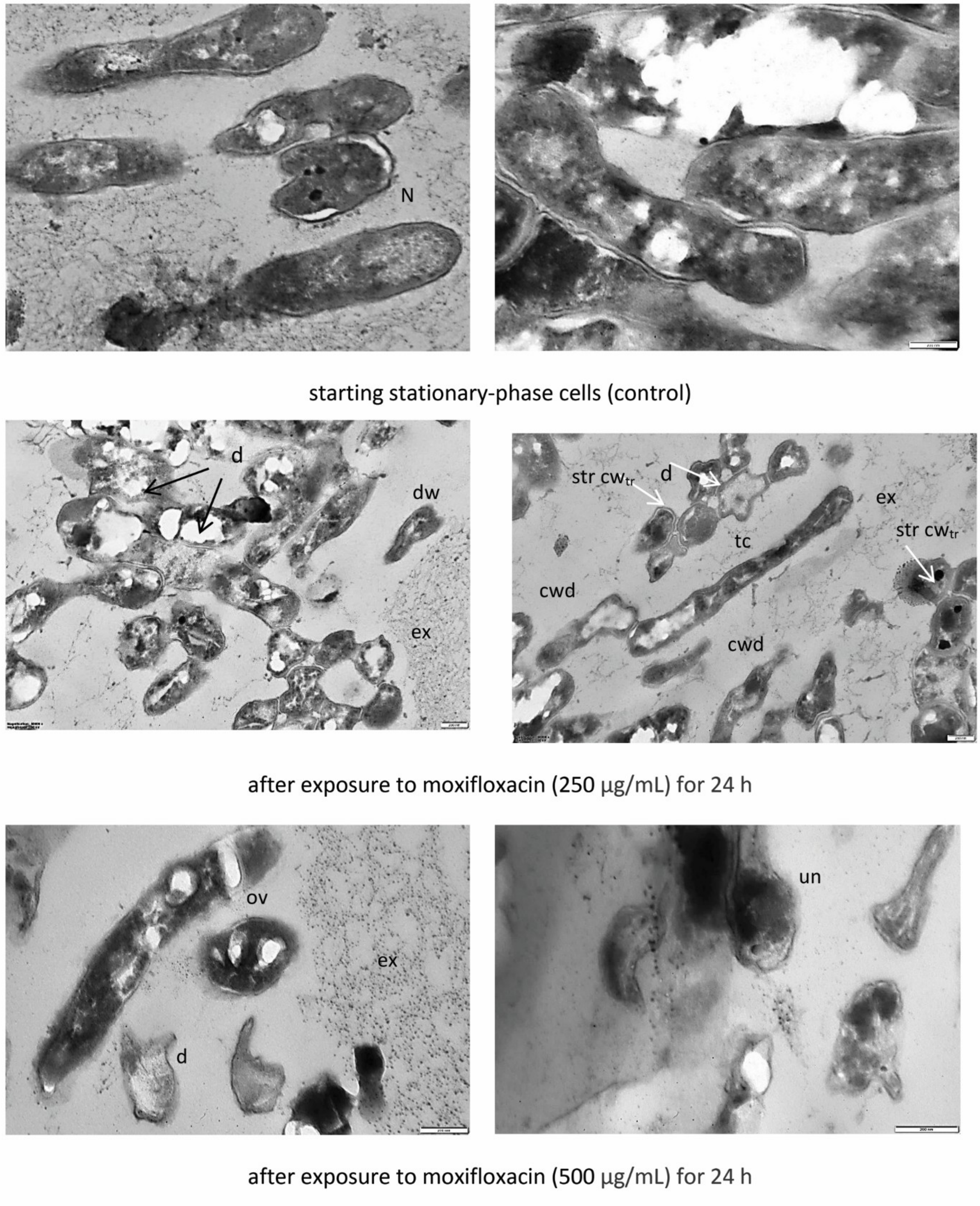

Supplementary Figure 7. Enlarged TEM images of thin sections for the control and moxifloxacin-treated *M. abscessus* cultures supplementary to **Figure 6**. Designations: cwd, cell-wall-deficient cells; d; destructed cells; dw, dwarf cells; ex, extracellular material; str cw$_{tr}$, stratified cell wall (transverse sections); ov, ovoid cells; tc, thin cells; un, unusual morphological type. Bars, 200 nm.

Supplementary Table 1. MIC values (µg/mL) of antibiotics for *M. abscessus* evaluated by the microtiter assay.

Resazurin reduction microtiter assay (REMA) was performed as previously described (Palomino *et al.*, 2002). Briefly, series of consequential twofold dilutions of antibiotics in Sauton medium with 10% ADC were added to wells. Each well contained also middle-logarithmic *M. abscessus* culture (10^5 CFU/mL). After incubation of the plates for 24 h at 37°C, resazurin (Merck, Germany) was added to wells at the concentration 0.025 mg/mL. Following overnight incubation at 37°C, fluorescence of resorufin, the resazurin metabolite, was measured using a Fluostar Omega plate monochromator spectrofluorimeter at excitation and emission wavelengths 544 nm and 590 nm, respectively. The MIC was determined as the least concentration preventing resazurin turnover from blue to pink as monitored visually and upon fluorescence measurements (Lechartier, Hartkoorn and Cole, 2012). MIC evaluation for each antibiotic was performed at least in two biological and two technical replicates.

Amikacin	8-16
Bedaquilin	2
Ciprofloxacin	8
Clofazimin	16
Linezolid	4
Moxifloxacin	2
Rifampicin	4

Supplementary Table 2. An amendment to **Supplementary Figure 5**. Relative content of elements as derived from EDX spectra of total examined fields for contol and experimental *M.abscessus* cultures exposed to moxifloxacin (500 µg/mL) for 24 h and 14 days.

Culture		Element, %							
		C*	O*	P	S	K	Ca	Mg	∑ other elements
Mid-logarithmic cultures	control	92.8±0.1	5.4±0.1	0.9±.0	0.2±0.0	0.3±0.0	0.00±0.0	0.2±0.0	0.02±0.0
	1 day	82.7±0.1	5.3±0.0	1.1±0.0	0.2±0.0	0.2±0.0	0.0±0.0	0.3±0.0	10.2±0.0
	14 days	26.8±0.8	8.4±0.1	0.0±0.0	0.2±0.0	0.1±0.0	12.9±0.0	1.8±0.0	49.8±0.3
Stationary-phase cultures	control	67.1±0.2	5.8±0.0	1.0±0.0	0.2±0.0	1,0±0.0	0.2±0.0	0.5±0.0	24.2±0.1
	1day	79.4±0.1	3.8±0.0	0.3±0.0	0.2±0.0	0.0±0.0	0.1±0.0	0.2±0.0	16.0±0.1
	14 days	61.4±0.4	6.4±0.1	0.0±0.0	0.2±0.0	0.0±0.0	3.1±0.0	0.4±0.0	28.5±0.2

*Signals for C and O are contributed by cells, their surrounding and Formvar (C-and O-containing) film on a grid.
